# A Computational model for compressed sensing RNAi cellular screening

**DOI:** 10.1186/1471-2105-13-337

**Published:** 2012-12-27

**Authors:** Hua Tan, Jing Fan, Jiguang Bao, Jennifer G Dy, Xiaobo Zhou

**Affiliations:** 1 School of Mathematical Sciences, Laboratory of Mathematics and Complex Systems, Ministry of Education, Beijing Normal University, Beijing, 100875, P. R. China; 2 Department of Radiology, The Methodist Hospital Research Institute (TMHRI), Weill Medical College of Cornell University, Houston, TX, 77030, U.S.A; 3 Department of Electrical and Computer Engineering, Northeastern University, Boston, MA, 02115, U.S.A

## Abstract

**Background:**

RNA interference (RNAi) becomes an increasingly important and effective genetic tool to study the function of target genes by suppressing specific genes of interest. This system approach helps identify signaling pathways and cellular phase types by tracking intensity and/or morphological changes of cells. The traditional RNAi screening scheme, in which one siRNA is designed to knockdown one specific mRNA target, needs a large library of siRNAs and turns out to be time-consuming and expensive.

**Results:**

In this paper, we propose a conceptual model, called compressed sensing RNAi (csRNAi), which employs a unique combination of group of small interfering RNAs (siRNAs) to knockdown a much larger size of genes. This strategy is based on the fact that one gene can be partially bound with several small interfering RNAs (siRNAs) and conversely, one siRNA can bind to a few genes with distinct binding affinity. This model constructs a multi-to-multi correspondence between siRNAs and their targets, with siRNAs much fewer than mRNA targets, compared with the conventional scheme. Mathematically this problem involves an underdetermined system of equations (linear or nonlinear), which is ill-posed in general. However, the recently developed compressed sensing (CS) theory can solve this problem. We present a mathematical model to describe the csRNAi system based on both CS theory and biological concerns. To build this model, we first search nucleotide motifs in a target gene set. Then we propose a machine learning based method to find the effective siRNAs with novel features, such as image features and speech features to describe an siRNA sequence. Numerical simulations show that we can reduce the siRNA library to one third of that in the conventional scheme. In addition, the features to describe siRNAs outperform the existing ones substantially.

**Conclusions:**

This csRNAi system is very promising in saving both time and cost for large-scale RNAi screening experiments which may benefit the biological research with respect to cellular processes and pathways.

## Background

RNA interference (RNAi) is an RNA-dependent gene silencing process that occurs in living cells and participates in controlling gene activation and how active these genes are [1]. The RNAi pathway is initiated by the enzyme dicer, which cleaves long double-stranded RNA (dsRNA) molecules (either endogenous or exogenous) into short fragments of ~20 nucleotides [2-4]. These short double-stranded fragments are called small interfering RNAs (siRNAs), which are sequences comprised by a four-alphabet, {A, U, G, C}. These letters tend to bind with one another in complementary base pairs: A with U, G with C, and vice versa. After the cleavage, siRNAs are then unbounded into two single strands, namely the passenger strand and the guide strand. The passenger strand is then degraded while the guide strand is involved in the formation of the so called RNA-induced silencing complex (RISC) [5]. At last, the siRNA attaches to its complementary target mRNA molecule and induces cleavage of the mRNA, thus prevents it from producing a protein [6]. This procedure is also termed gene knockdown [7].

The selective and robust knockdown effect of RNAi on gene expression makes it a significant research tool that enables specific repression of interested genes [8]. RNAi is widely used for large-scale screening in which particular genes in a certain cell line are silenced at a systems level. This large scale approach is very helpful to identify the causal components for a particular cellular process or an event such as cell division [9]. As a result, plenty of work to design effective and specific siRNAs has been provoked [10-14]. Among these, the most well known ones were introduced by Huesken et al. and Reynolds et al. Huesken et al. adopted an artificial neural network based algorithm called BIOPREDsi to design a genome-wide siRNA library [15]; and Reynolds et al. identified eight characteristics (criteria) associated with siRNAs to design siRNAs in a rational way [16]. The previous work advanced the efficient identification and design of effective siRNAs and consequently, improved the gene knockdown (or RNAi) experimentation both in specificity and efficiency. However, the largest gene knockdown experiments performed so far have used multiple siRNAs per gene [9,17].

In a genome-wide screening, biologists are able to observe phenotypes with respect to affected cytoskeletal organization and/or cell shape. Changes in intensity, cell size and other morphological features can be quantified to indicate the effectiveness of RNAi procedures and this is well established [18]. The measurement on RNAi success can always be modified to binary indictors which equals one if RNAi works and zero otherwise. And these procedures are fully automated [18]. Thus for an siRNA screening, we can acquire a binary measurement from the wells on the array. However, from a systems level, the specified one-to-one relationship between siRNA and target gene hinders a high-throughput screening (HTS) of functional genes in a genome-wide scope.

To overcome this burden for designing a large scale RNAi-based screening of functional genes with better efficiency, we propose this conceptual model, the compressed sensing RNAi (csRNAi), based on the fact that a target-complementary siRNA can simultaneously target at other mRNAs containing sequence segments that are partially complementary to it [19]. The main idea is, unlike the traditional scheme in which one siRNA knockdown only one gene (by cleaving or repressing its transcribed mRNA), we employ a unique combination of group identifier siRNAs to knockdown a group of genes and we expect the number of siRNAs to be much smaller than that of targeted genes. In this model, the group identifier siRNA is a certain motif locating at a certain group of genes. In mathematical language, group identifier siRNAs constitute a basis which spans a space containing a larger number of genes than the number of instances in the basis. However, the off-target effect (OTE) [20] which refers to the scenario that one siRNA can knockdown more than one gene, is very undesirable for specific knockdown. Although one group identifier is located at a collection of genes, which suggests it can knockdown several genes, it is different from OTE in that the group knockdown is specific and consequently does not spread to other genes to be considered as OTE, thanks to the limited locations as a unique combination of the group identifier siRNAs.

From a signal processing prospective, the compressed sensing (CS) is a technique for reconstructing a signal of length *n* (generally very large) that is *K*-sparse from only *m* (*m* « *n*) linear measurements of the signal, using the prior knowledge that the original signal is sparse or compressible. This means that there is some redundancy in the most interesting signals. Here *K*-sparse means there is at most *K* nonzero components in the vector of a signal. This is consistent well with the group testing scheme in that eventually few genes are effectively knockdown in a real RNAi experiment. We will explain the mathematical model of our csRNAi strategy and its implementation in the section of *Methods*. Note again, the cross-hybridization property of an siRNA with several mRNA targets, not just one, is crucial for applying CS principles.

In addition to proposing the csRNAi concept, we also improve current siRNA design by introducing a much larger and more conclusive feature space, as well as a machine learning scheme. Concretely, we first perform motif search within a group of genes, and consider these motifs as candidate group identifiers; a classifier is then built to identify the real group identifiers (true siRNAs) from the candidates, by employing our proposed image and speech features along with other traditional descriptive traits. Furthermore, the position specific feature (PSF) exaction strategy does not depend on any specific data set and is able to detect both desired and undesired sequence content, with detail documented in the *Methods* section and the Additional file 1. The classifier identifies sequences with larger probability to be siRNAs, which is a group identifier as well. By comparing with existing rules and methods [10,19,21], we observe better performance of our siRNA prediction method.

In summary, we propose a novel compressed sensing RNAi (csRNAi) cellular screening system, in both conceptual and bench experiment level. We present a small-scale csRNAi example served as a proof-of-concept, to reveal how the RNAi experiments coordinates with the prerequisites of the compressed sensing theory. We also show by numerical simulations that about one third of traditionally necessary siRNAs is sufficient to reconstruct the original sparse signal, i.e., to identify the functional genes accurately; and this compression ratio is expected to be further improved in larger scale screening in real experiments.

## Methods

### The compressed sensing RNAi (csRNAi) model

**Figure 1:**
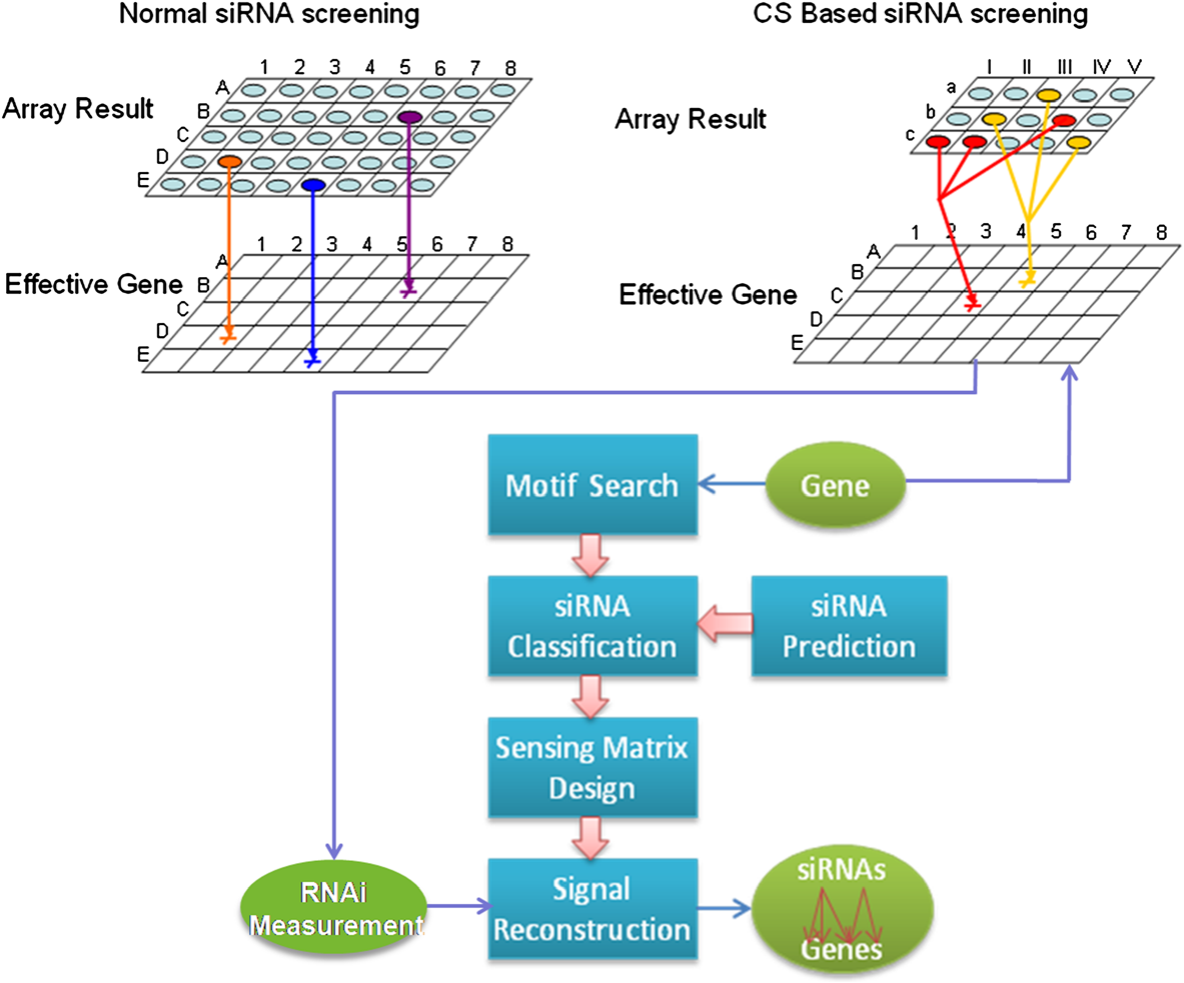
**Illustration of conventional and compressed sensing RNAi (csRNAi) screening along with the implementation of the csRNAi scheme.** For conventional RNAi screening, one siRNA is designed to respond to one target gene (upper left); for compressed sensing based RNAi screening, one siRNA can bind to several target genes simultaneously and vise visa (upper right). In the implementation flowchart (lower panel), the blue rectangles represent processing procedures and green ellipses stand for input and output. The readout of the RNAi experiment serves as an input of the signal reconstruction step.

This csRNAi model (group knockdown strategy) is illustrated by Figure 1 in comparison with its traditional counterpart. The upper left panel shows the conventional (normal) RNAi screening, i.e., each spot in the microarray has an RNA fragment that serves as a unique identifier of only one target gene; while the upper right panel refers to our proposed csRNAi scheme, in which the same target gene group can be knockdown with an siRNA pool of much smaller size. The lower panel depicts the implementation flowchart of the csRNAi model, which includes four progressive steps: sequence motif search, effective siRNA selection, compressed sensing (CS) matrix design and signal reconstruction. Of note, the first three steps can be conducted via *in silico* method, while the fourth step (signal reconstruction) needs the RNAi profile measurement as input. The next subsections provide more details about the implementation.

We stress that the readout of each probe is a probabilistic combination of interfering outcome of the siRNA with its target genes. Here the probabilities reflect the strength or effectiveness of the siRNA to knockdown associated genes. Depending on particular applications, the readout could be any measurable signal such as cell phenotype change, fluorescence intensity shift or variation of mRNA expression level that can be evaluated by a numerical quantity. These measurements can be obtained via standard experiment methods. For a detailed description of a bench experiment about the CS based microarray configuration and implementation, please refer to [22]. The real challenge is, however, how to infer the knocked-out genes, or interfering profile, from so few available observations (readouts in the wells). And we claim that this is exactly what the compressed sensing theory answers.

### Sequence motif search

Nucleotide sequence motifs simultaneously located at several genes are usually of biological significance [23]. These shared patterns therefore have the potential to act as binding sites of siRNAs, and we will confirm its role as an siRNA by further operation in next section. We search sequence motifs using the online-accessible software MEME (Multiple EM for Motif Elicitation), which is one of the most widely used tools for identifying motif signals in DNA and protein sequences [24]. Although RNA interference is mediated by 21- and 22-nucleotide RNAs [4], biochemical studies reported that guide-strand position 1 and the nucleotides at the 3’ overhang (position 20 and 21) have little, if any, contribution to the specificity of target recognition. Furthermore, mismatches near the 5’and 3’ends can be tolerated provided other positions remain unchanged [19,25]. Hence we only search for motifs with length of 19bp to be consistent with the public available siRNA data and to accelerate the procedure.

**Figure 2:**
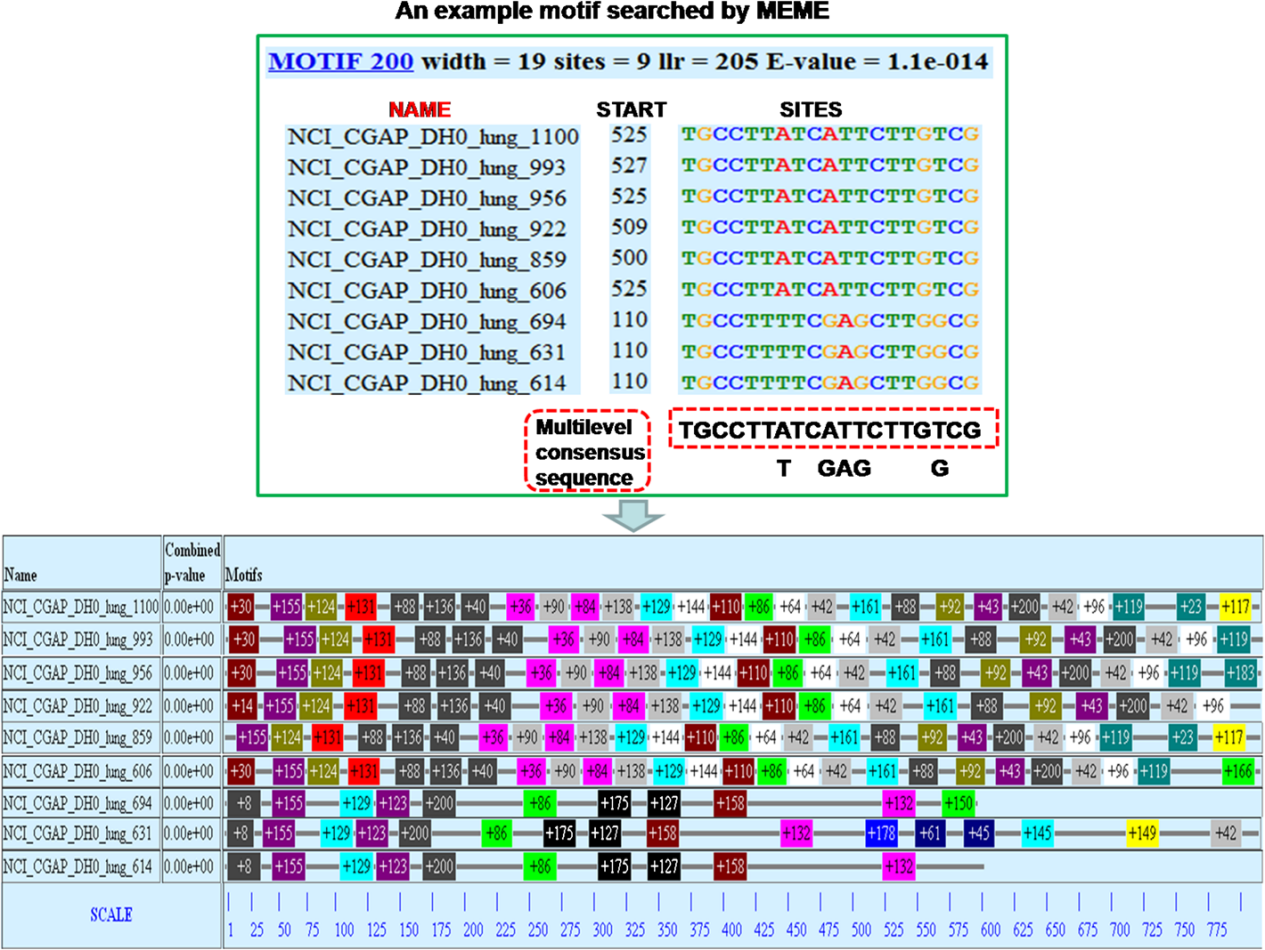
**An example motif searched by the software MEME.** The top panel illustrates an example motif by a summary line and the occurencs of the motif in 9 target genes sorted by p-value. The rectangular red box shows the maximum consensus sequence (MCS, see text) of the motif. The lower panel summarizes all motif occurrences of each involved target gene.

Figure 2 shows an example motif identified by MEME, along with a summary of the motif occurrences of its involved target genes. Here E-value is the statistical significance of the motif. Specifically, E-value is an estimation of the expected number of motifs with the given log likelihood ratio (llr) or higher, and with the same width and number of occurrences, that one would find in a similarly sized set of random sequences. Therefore the motifs searched by MEME bear significant potential to be group identifier siRNA candidates. A multilevel consensus sequence shows the most conserved letter(s) at each motif position. We choose the top level as the candidate siRNA and term it as maximum consensus sequence (MCS) for short. Consequently, the MCS is a consensus sequence with each nucleotide appearing the most frequently at that position of all occurrences. Note that the MCS may fail to be exactly complementary to some target genes, but as we have mentioned above, it is the situation we need, since one siRNA is expected to be base pair to several genes simultaneously, with distinct degrees of specificity. Finally, if motif search is based on mRNAs, each motif should be translated into its complementary sequence to be an siRNA that targets the mRNA from which the motif was found. In this work, we search motifs from a dataset of cDNAs with respect to human lung cancer and hence it is unnecessary to perform this transformation.

### Effective siRNA selection

In the previous part, we identified several MCSs as candidates of group identifiers. To be a valid group identifier, a sequence should not only be a shared motif, but also an effective siRNA. Therefore, prediction of high confident siRNAs is a crucial part of the proposed csRNAi system. Instead of randomly picking some sequences and verifying them biologically, which suffers low efficiency and high cost, biologists and bioinformatics researchers seek for an accurate prediction method of siRNAs. We propose a machine learning based method, which introduces novel features in addition to the current popular prediction rules and features. These novel descriptors include 1) image features, in which we convert a gene sequence into an image and 2) speech features in which we consider that each element in the siRNA sequence is not entirely independent but with some semantic relevance. We also improve the existing position specific features (PSF) [11] to enable our method to adapt to more general scenarios. Our strategy to extract PSF features is flexible in the sense that it derives distinct rules according to different training inputs. Moreover, the proposed method considers both desired and undesired nucleotide content for a specific sequence position. Widely used features such as thermodynamic (TD) features, *N*-Gram and *N*-GSK (general string kernel) features [21] and some coding features are studied individually and grouped altogether. We also focus on the 2 base-pair (bp) patterns to extract the sequence position specified feature (SPSF) and position coding (PC) features. The data used in this study is from Huesken et al. 2005, which contains 2431 siRNAs as well as their interference activities. The interference activity is an indicator of the effectiveness of the siRNA. We build three data sets with different cut-off values of interference activities. Details about the data and how we extract the mentioned features for a given sequences are provided in Additional file 1. The following paragraphs in this subsection emphasize the novelty and advantage of these proposed features.

**Figure 3:**
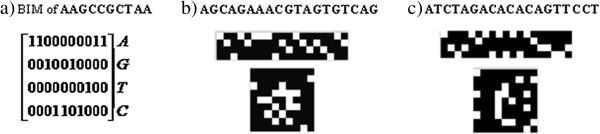
**Image representation of a genetic sequence. a**). BIM representation of AAGCCGCTAA; **b**). BIM (top) and image (bottom) representation of a positive training sample in data set 2; **c**). BIM (top) and image (bottom) representation of a negative training sample in data set 2.

We first describe how an alphabetic sequence can be treated as an image and what types of features can be employed to depict a gene sequence as an image. If the gene sequence is represented by a binary indicator sequence, this will transform the one-dimensional gene into a two-dimensional image. For example, the sequence AAGCCGCTAA of length 10 can be expressed as a 10-by-4 binary indicator matrix (BIM) with the rows representing A, G, T and C sequentially, as illustrated in Figure 3.

If considered as an image, the binary indicator matrix of an siRNA behaves as random noise because of sparsity. However, if we can transform the BIM into formats containing some shape information, it may be helpful to extract features like texture and moment features. Inspired by cell image processing, we prefer that the transformed BIM possesses rounded shape similar to the cells. Therefore, we separate a round disk into *N* slides with *N* spokes with equal angle, where *N* is the length of sequence, and put a four character length code on each slide. Then we fit the disk into a 9-by-9 matrix centered at pixel (5, 5) where each element stands for a pixel. Figure 3 shows an example of sequence image, more detail please refer to Additional file 1.

By representing each sequence as an image, we follow the similar strategy in cell image classification to extract features [26]. These image features include Gabor wavelet feature, Haralick co-occurrence texture feature and Zernike moment feature. To summarize, we extract 132 features in this part, as explained in the Additional file 1.

Genomic sequences are comprised by an alphabet with four characters, A, G, T (U for RNA) and C. In speech signal processing and recognition, the human voice is converted to machine-readable signals, for example, a binary code for a string of a certain alphabet. We intend to understand the genomic sequence with well-established speech recognition technique based on the facts that 1) both of speech signal and siRNA, more generally, DNAs or RNA sequences show a wave signature and 2) if digitalized by some coding methods, gene sequences can be considered as signals and processed with signal processing tools, especially in the transformed domain; 3) speech recognition techniques are useful because the linear prediction property exists in some positions of a gene sequence.

The alphabetic sequences of the siRNAs are digitalized by the coding book: the code for A is 1000, for G is 0100, for T is 0010 and for C is 0001. Therefore, a 19 bp-length siRNA sequence can be signified by a binary digit sequence (Additional file 1). Among various speech recognition feature groups, linear prediction coefficients (LPC), mel-frequency cepstrum coefficients (MFCCs) and wavelet features have been proved to be the most successful features for speech recognition [27]. Linear prediction is based on the auto-regression model to determine a set of parameters or predictor coefficients that minimize the mean square errors between the actual and predicted signals. Although widely used, however, LPC is limited by the nature of linearity. MFCCs are applied in speech/speaker recognition and increasingly in music information retrieval [28]. The perceptual linear predictive (PLP) analysis considers psychophysics of hearing together with linear prediction method. The linear prediction method can be improved by adding the critical-band spectral resolution, the equal-loudness curve, and the intensity-loudness power law, finally approximated by an autoregressive all-pole model. It suppresses the slowly varying component in each frequency channel and enables the estimate to be less sensitive to slow variation noise in the short-term spectrum. In our implementation, the total number of features in this part is 199, as listed in Table 1. The Additional file 1 provide detailed explanation about the derivation of these features and parameter settings.

**Table 1:** A summary of all the features originally extracted

**Feature class**	**Feature description**	**#**	**Feature class**	**Feature description**	**#**
**Position Specific Feature (PSF)**	Important position content selected according to P-value	20	**Sequence Position Specific Feature (SPSF)**	Important sequence position content selected by P-value	16
**N-Gram Feature (N-Gram)**	N-length sequence gram features: occurrence of the subsequence	336	**N-GSK Feature (N-GSK)**	N-length sequence GSK features: occurrence of the subsequence	336
**Thermodynamic Feature**[29]	Gibbs free energy calculated from Identical Nearest Neighbors model	21	**Image Feature (Image)**	Texture and moments features extracted from coded sequence	364
**Position Composition Feature (PC)**	A coding method without statistical process	132	**Speech Feature (Speech)**	MFCC, LPC, PLP extracted from the sequence	199

We obtain a large feature space of dimension 1424 which is quite likely to be over-fitted. To solve this issue, we perform feature selection algorithm called SVM-RFE (support vector machine-recursive feature elimination) on each feature group, and also on the combined feature space. By this method we substantially shrink the dimension of the feature space (Table 2, see also Additional file 1: Table S14-Additional file 1: Table S15) while improve the cross-validation (CV) accuracy significantly (Additional file 1: Figure S5).

**Table 2:** Number of features with and without feature selection

**Feature Groups**	**PSF**	**SPSF**	**TD**	**N-Gram**	**N-GSK**	**PC**	**Image**	**Speech**	**All**
**W/O Feature Selection**	20	16	21	336	336	364	132	199	1424
**FS individually**	18	16	21	55	30	49	34	75	298
**FS combined together**	6	4	1	56	13	39	8	16	143
**Proportion**	30%	25%	4.8%	16.7%	3.9%	10.7%	6.1%	8.0%	10.0%

Eventually the classifier consists of 143 descriptive traits after the SVM-RFE procedure. We adopt SVM-light [30] as the classification tool and train it with a training data set which contains 2431 biologically confirmed siRNAs (2182 training and 249 testing records) along with their normalized interference activities [15].

### Compressed sensing matrix design

**Figure 4:**
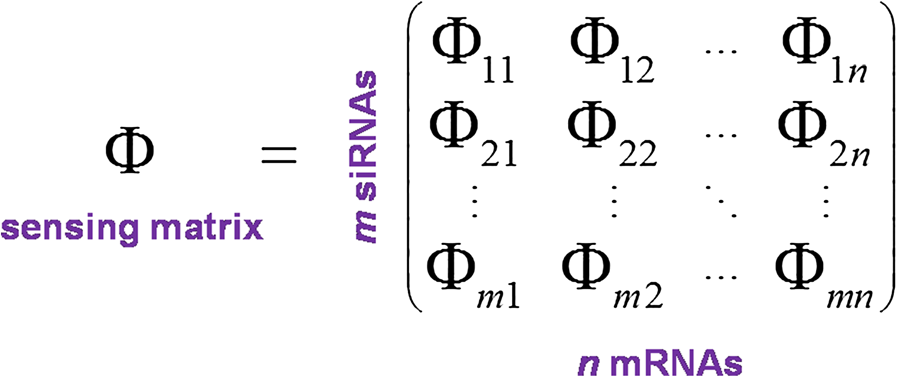
**Schematic of the sensing matrix in relation to number of siRNA and mRNAs.** The compressed sensing matrix has much less rows than columns, i.e., *m* ≪ *n*.

Once we obtain the siRNAs, their target genes can be derived based on the motif search result. Considering the example shown in Figure 2, MOTIF 200 is found to locate in nine genes (IDs: 1100, 993, 956, 922, 859, 606, 694, 631, 614) with a sufficiently small E-value 1.1e-014. On the other hand, these genes may contain other motifs as candidate group identifiers. The candidate siRNAs and their putative target genes consequently form a multi-to-multi correspondence, which fits to a network structure. Although rarely the case, for genes which share exactly same motif group, we randomly choose one of them and discard the remaining, because genes can eventually be identified only if a group of siRNAs uniquely encodes one gene. Then we mathematically represent the network as a sensing matrix **Φ** whose rows stand for siRNAs and columns refer to mRNAs (targets) (Figure 4, see also [22]).

The entries of the sensing matrix are arranged in the manner that if motif *i* does not respond to gene *j* according to the motif search, then Φ_*ij*_=0; otherwise, we endow Φ_*ij*_ a positive value that denotes the combining affinity between motif *i* and gene *j*. Although researchers proposed several parameters to configure the nonzero affinity value, Xu et al. [31] achieved promising performance by using percent identity (percentage of matched bases in the aligned region) as the single parameter. Hence in this study, we consider the identity percent alone and implement the configuration row by row in the following procedure: for each motif *i*, which corresponds to the *i*^*th*^ row of the matrix, we select the index of its potential targets from the motif search result, and assign a nonzero probability for the corresponding columns within the current row. This probability is calculated based on its degree of consistence with the maximum consensus sequence (MCS). We compute the probability as the number of bases identical to the MCS divided by the width of the motif (i.e., percent identity). Taking Figure 2 for example again, the columns with respect to targets indexed by (1100, 993, 956, 922, 859, 606, 694, 631, 614) are assigned with probability $\left(1,1,1,1,1,1,\frac{14}{19},\frac{14}{19},\frac{14}{19}\right)$ respectively. The remaining entries in this row are assigned 0 to indicate that there is no or very low hybridization affinity between the corresponding targets and Motif 200. Finally, we regulate columns of **Φ** into unit *l*_2_ -norm, which is a prerequisite of compressed sensing theory and will be further explained.

Of note, our scheme of CS matrix design implicitly assumes the binding affinity between an siRNA and its target gene depends solely on the sequence match degree (percent identity). This simplification is beneficial for the implementation of the whole csRNAi system, since it avoids complicated algorithms for the design of probe sequences, as in [22].

### Signal reconstruction

In RNAi screening, cellular morphological changes could be quantified as the effectiveness of gene knockdown. In a large-scale (e.g., genome-wide) RNAi experiment, not all potential target genes can be recognized as knockdown genes in the screening based on the final cell phenotype alterations. To find the small part of genes that really contributes to phenotype change is biologically significant. Since the phenotype alterations can be obtained by running the bench experiment in the lab, the functional genes can be identified with the help of compressed sensing RNAi (csRNAi) strategy.

To be concrete, our goal here is to predict the silencing profile of *n* genes from only *m* (*m* « *n*) readouts of measurement change in *m* wells (Figure 1). Mathematically, we denote siRNAs by an *m*-dimensional vector **y** = (*y*_1_, *y*_2_, ⋯, *y*_*m*_)^'^ and target genes by an *n*-dimensional vector **x** = (*x*_1_, *x*_2_, ⋯, *x*_*n*_)^'^ . Their correspondence is described by the *m* × *n* sensing matrix **Φ** as introduced in the previous paragraph. With this mathematical notation, the task is to uniquely solve the following system of linear equations given the measurement vector **y** and sensing matrix **Φ**:

(1) y=Φx

This seems impossible in general because it is underdetermined, i.e., there are fewer equations than the number of unknowns. However, the recently developed compressed sensing (CS) theory guarantees that there exists unique solution to (1) with some preconditions.

To perfectly reconstruct the signal in the underdetermined condition, there are two critical prerequisites in compressed sensing theory [32]: (1) the sparsity condition: the signal vector **x** to be sensed should be sparse enough. This means that **x** has a very limited number of nonzero components, although we do not know a priori which of them are nonzero; (2) the incoherence condition: the rows of the sensing matrix **Φ** are sufficiently incoherent. Incoherence is achieved if **Φ** satisfies the so-called Restricted Isometry Property (RIP). For example, random matrices with independent identically distributed (i.i.d.) entries, such as Gaussian or ±1 binary entries exhibit a very low coherence [32,33]. Furthermore, in most practical situations, we cannot assume that **Φx** is known with arbitrary precision. Therefore, we consider some unknown perturbation instead of an exact case and modify equation (1) accordingly to obtain

(2) y=Φx+e

where **e** = (*e*_1_, *e*_2_, ⋯, *e*_*m*_)^'^ is some unknown perturbation satisfying $||e|{|}_{l_{2}}\leq \varepsilon$.

Next, we present the most important result recently developed in compressed sensing theory and how it applies to the proposed csRNAi system, or how a biological question can be answered by a modeling scheme. Let **Φ**_**T**_, **T** ⊂ {1, ⋯, *n*} be the *m*×|**T**| submatrix obtained by extracting the columns of **Φ** indexed by **T**. Define the *S*-restricted isometry constant *δ*_*s*_ of **Φ** which is the smallest quantity such that

(3) $$\left(1-\delta_{S}\right)||c|{|}_{l_{2}}^{2}\leq ||\Phi_{T}c|{|}_{l_{2}}^{2}\leq \left(1+\delta_{S}\right)||c|{|}_{l_{2}}^{2}$$

holds for all subsets of **T** with |**T**|≤*S* and coefficient sequences(*C*_*j*_)_*j*∈**T**_.

Suppose **x**_0_ is a sparse signal, which indicates the support T_0_ = {*t*: **x**_0_(*t*) ≠ 0} of **x**_0_ is assumed to have small cardinality. Consider the convex program searching, among all signals consistent with the data **y**, for that with minimum *l*_1_-norm

(4) $$\left(P_{1}\right)\begin{array}{ccc} & & \min||x|{|}_{l_{1}}\begin{array}{cc} & s.t. \end{array} \end{array}\begin{array}{rr} & ||\mathrm{\Phi x}-y|{|}_{l_{2}}\leq \varepsilon \end{array}$$

The following theorem shows that the solution to (*P*_1_) recovers an unknown sparse signal with an error at most proportional to the noise level.

**Theorem 1** (Candes-Romberg-Tao, 2006) Let S be such that *δ*_3*Ѕ*_+3*δ*_4*Ѕ*_<2. Then for any signal **x**_0_ supported on **T**_0_ with |**T**_0_|≤*Ѕ* and any perturbation **e** with $||e|{|}_{l_{2}}\leq \varepsilon$, the solution **x**^#^ to (*P*_1_) obeys

(5) $$||x^{\#}-x_{0}|{|}_{l_{2}}\leq C_{S}\cdot \varepsilon$$

Here the constant *C*_*S*_ only depends on *δ*_4*S*_.

Interested readers are referred to [34] for detail of the proof of Theorem 1. This theorem transforms the following *l*_0_-regularization problem (*P*_0_) which requires combinatorial optimization into a more tractable convex programming problem (*P*_1_), as shown in (4).

(6) $$\left(P_{0}\right)\begin{array}{ccc} & & \min||x|{|}_{l_{0}}\begin{array}{cc} & s.t. \end{array} \end{array}\begin{array}{rr} & ||\mathrm{\Phi x}-y|{|}_{l_{2}}\leq \varepsilon \end{array}$$

The restricted isometry property (RIP) imposed on **Φ** essentially requires that every set of columns with cardinality less than *S* approximately behaves as an orthonormal system. In practice, we evaluate the behavior of **Φ** by the absolute maximal inner product *M*_Φ_ among all the inner products of its column pairs:

(7) $$M_{\Phi }=\max\left\{|<\Phi_{i},\Phi_{j}>|,1\leq i,j\leq n,i\neq j\right\}$$

**Figure 5:**
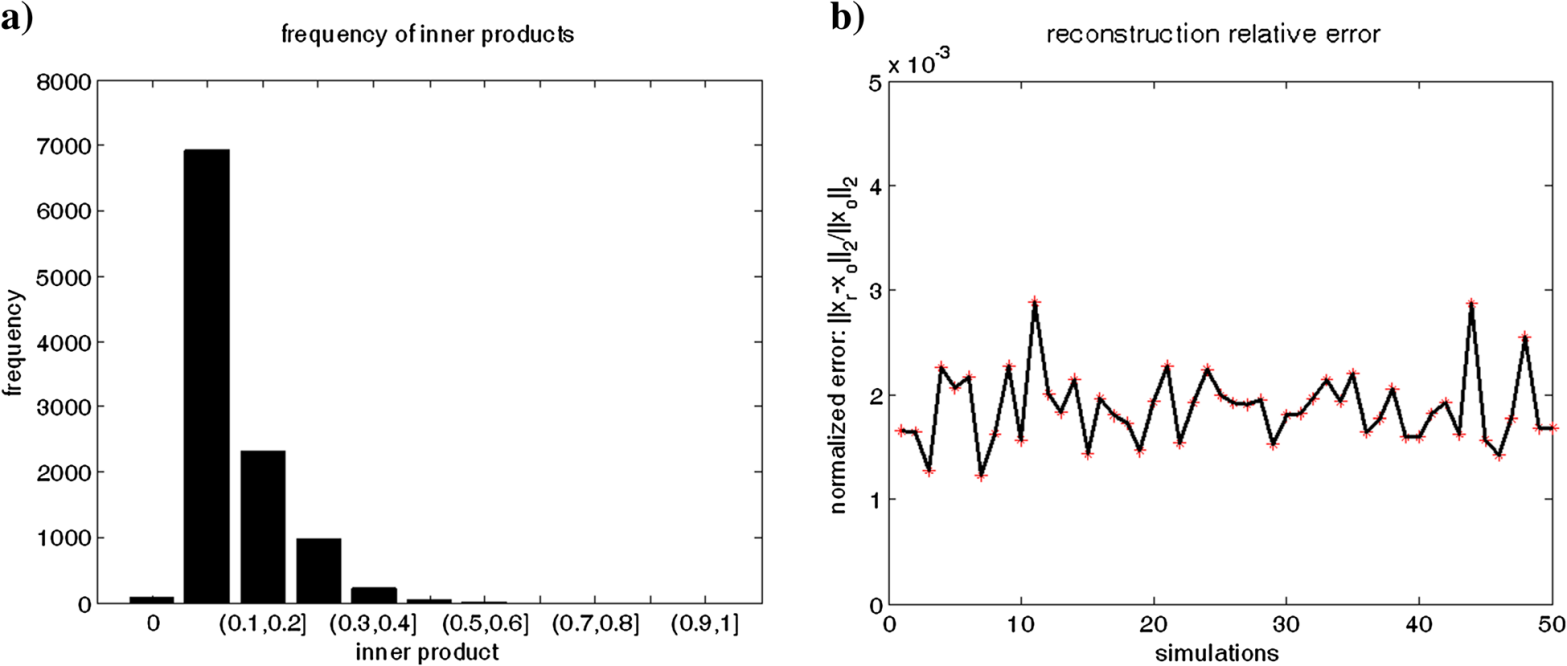
**An ideal example based on Donoho’s result. a**). Illustration of the frequency of the 10585 inner products. All of them are less than 0.6 and those locating between 0.1 and 0.2 take an over-whelming part. **b**). Reconstruct simulation for 50 times, with a noise ε∈ *N*(0,0.001), where x_0_ and x_r_ are original and reconstructed signal respectively. The normalized errors are bounded invariably by 0.3%.

The smaller the *M*_Φ_, the better the system behaves. We give an ideal example according to one strategy provided by Donoho [35], where the sensing matrix **Φ** is generated by concatenating several orthonormal bases. Figure 5 illustrates the frequency distribution of inner products of **Φ** and the performance of signal reconstruction, where **Φ** is a 48×146 matrix whose columns are a concatenation of several orthonormal bases of **R**^48^_,_ and *M*_Φ_=0.5052.

## Results

**Figure 6:**
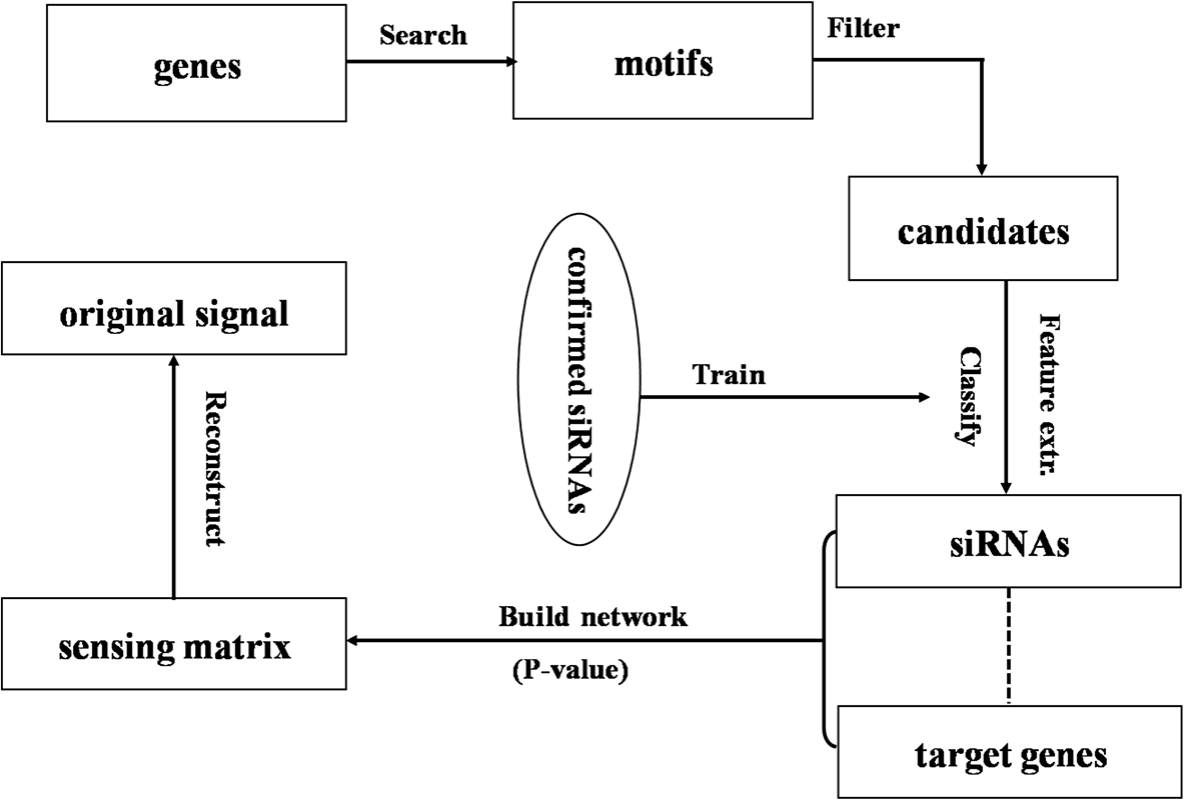
**Flowchart of the numerical experiment.** Illustration of how the numerical experiment is implemented following the four progressive steps presented in *Methods* section.

To clearly demonstrate the idea of CS-based RNA interfering, we present a numerical experiment of a small-scale csRNAi system. The detail of how we implemented each step and how we obtained the related data is elucidated in *Methods*. Figure 6 illustrates the flowchart of the numerical experiment. We first identified 200 motifs (indexed by *s1* to *s200* in this work) of 19 nucleotides as candidate group identifiers from 600 EST (expressed sequence tag, a short sub-sequence of a cDNA sequence) sequences (indexed by *g501* to *g1100*). Figure 2 illustrates an identified motif with its associated target genes, and Additional file 1: Figure S7 provides more examples. The abovementioned EST dataset (with 600 elements) is a part of a cDNA library (of UniGene) NCI_CGAP_DHO (http://cgap.nci.nih.gov/Tissues/LibInfo?ORG=Hs&LID=26664**)** which contains a total of 4647 EST sequences related to VS-8 cell line from Metastatic Chondrosarcoma in lung. We chose the shorter EST sequences instead of the original whole cDNAs to significantly shorten the motif search time without information loss, since the EST sequence contains enough information to permit the design of precise probes for DNA microarrays that then can be used to determine the gene expression [36]. We performed the motif search using the software MEME as mentioned and extracted the 200 maximum consensus sequences (MCS) from the search result. We discarded the MCSs with the composition like AA…AC because almost all the chosen 600 genes start with this pattern and intuitively, the software would invariably identify such kind of motifs, but apparently they are not good group identifiers.

After previous processing, we obtained 176 siRNA candidates as group identifiers. Since we intend to propose a csRNAi system in this paper, for the siRNA prediction, we only present key results here. The details are provided in the Additional file 1, in which we analyzed the prediction results based on each feature group independently with and without feature selection. Then all the features were combined and feature selection was applied. The classification results for each feature group were listed in Additional file 1: Table S5, Additional file 1: Table S6, Additional file 1: Table S7, Additional file 1: Table S8, Additional file 1: Table S9, Additional file 1: Table S10, Additional file 1: Table S11 and Additional file 1: Table S12, individually. Additional file 1: Figure S5 illustrates the prediction accuracy with and without SVM-RFE on data set 2. There are totally 1424 features if combining the entire feature group together. Feature selection keeps 143 features, which is ten percent of its original size. The cross validation accuracy can reach as high as 88%, which improves by 21.5% by discarding about one thousand features. Table 2 lists the numbers of features of each group that comprise the final selected feature space. These proportions demonstrate the importance of different feature groups. For example, although the PSF has merely 20 features, 30% of them are kept, which is three times of the average ratio 10%. Additional file 1: Figure S4 compares the prediction accuracy among the position specified rules derived by literature and our method. It can be seen from the figure that our rules consistently yield higher prediction accuracy in all cases.

**Figure 7:**
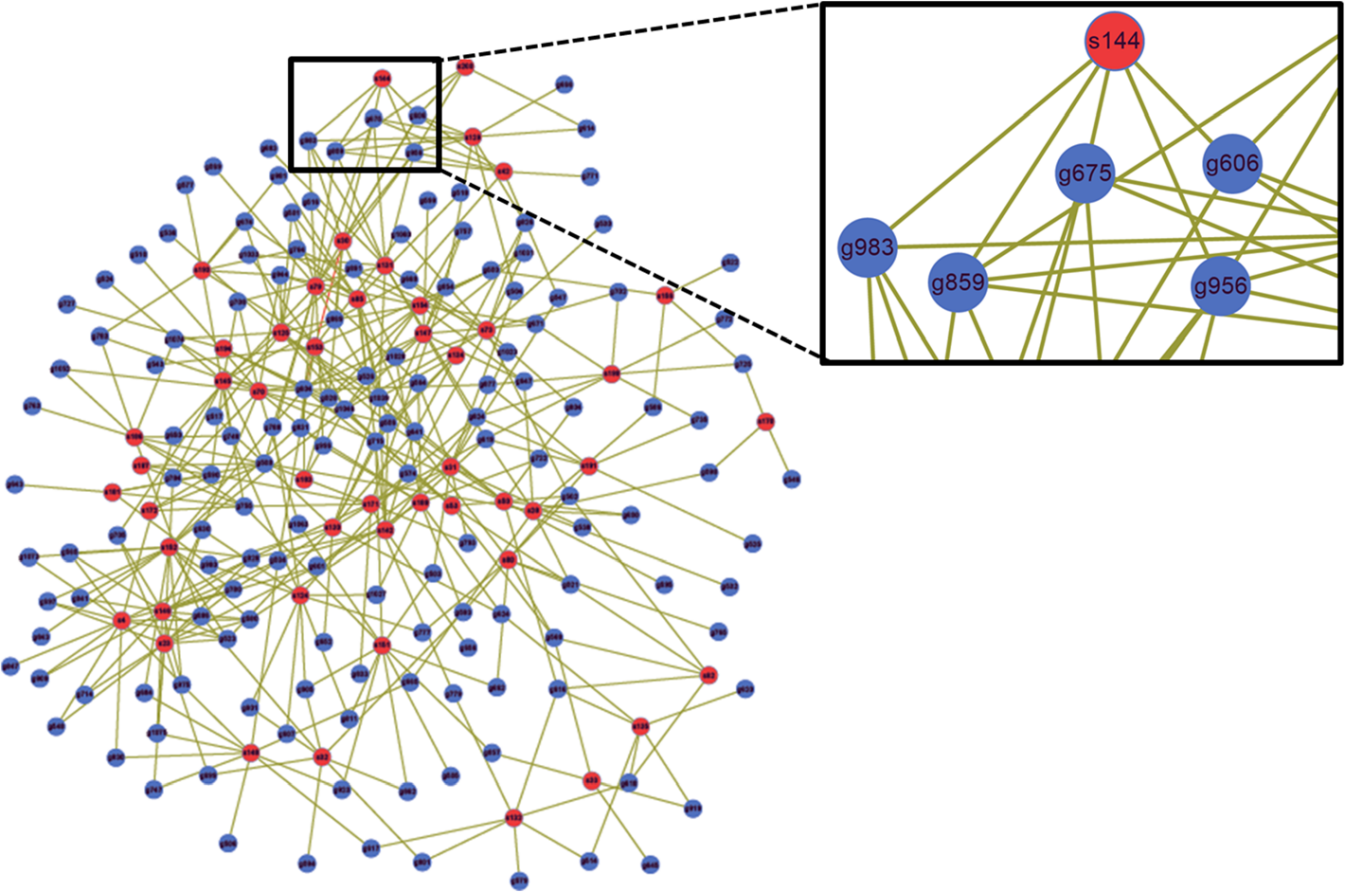
**Multi-to-multi siRNA-mRNA network.** The network is composed of 48 group identifier siRNAs (red balls) and 146 putative target genes (blue balls). The knockdown relationship is illustrated by solid lines connecting siRNAs and genes, and the index of each object is displayed on the node, as shown in the zoomed-in sub-graph.

We employed SVM-light to categorize the candidates into 49 siRNAs and 127 non-siRNAs using the previously mentioned classifier and feature space. These 49 siRNAs correspond to 147 target genes in a multi-to-multi manner. By checking the correspondence we found that motif 164 only targeted at gene 541 and vice versa, hence we discarded this 1-to-1 correspondence and eventually obtained a 48×146 network. We drew this figure of network using Cytoscape, an open source platform for complex-network analysis and visualization [37]. The network is shown in Figure 7 (see Additional file 1: Figure S8 for the heatmap visualization), in which the red balls stand for siRNAs and the blue ones represent target genes. The connection between gene and siRNA indicates the knockdown relationship. We present details of all the siRNAs and target genes involved in this network in Additional file 1: Table S17 and Additional file 1: Table S18 respectively.

Then we transformed the network into a sensing matrix **Φ** following the step described in the *Methods* section. Among the *C*_146_^2^ = 10585 inner products of columns of Φ, 9170 are 0 and those with a smaller value than 0.6 take an overwhelming part (data not shown). This implies that to a large extent, each subset of columns of **Φ** behaves like an orthonormal system. We first randomly generated the original signal with each component **x**_*o*_(*t*)∈*U*(0,1), *t* = 1, 2, ⋯, *n* and the Gauss white noise with each element **e**(*t*)∈*N*(0,*σ*), *t* = 1, 2, ⋯, *m*. To obtain a *K*-sparse signal, we set all but the largest *K* components of **x**_*o*_ to zero. Consequently we got the hypothesized measurement **y**= **Φx**_o_ + **e**, where **x**_*o*_ has been calibrated to *K*-sparse already. To emphasize again, in an actual RNAi experiment, the measurement vector **y** could be a continuous or binary vector to indicate the averaged cell intensity, cell size or other morphological changes before and after the application of RNAi. It also could be a certain biochemical signal change at the molecular level, to name but a few, fluorescence intensity shift or variation of mRNA expression level, depending on the specificity of the experiment. To confirm the feasibility of this strategy mathematically, we assume this measurement is available to us and the simulation is performed with different **y** s. Therefore the task eventually became to reconstruct the signal **x**_*o*_ given **y, Φ, e**, and to compare the reconstructed signal **x**_*r*_with the original one **x**_*o*_. We applied Hale’s open codes [38] to implement the *l*_1_-regularization problem (*P*_1_).

**Figure 8:**
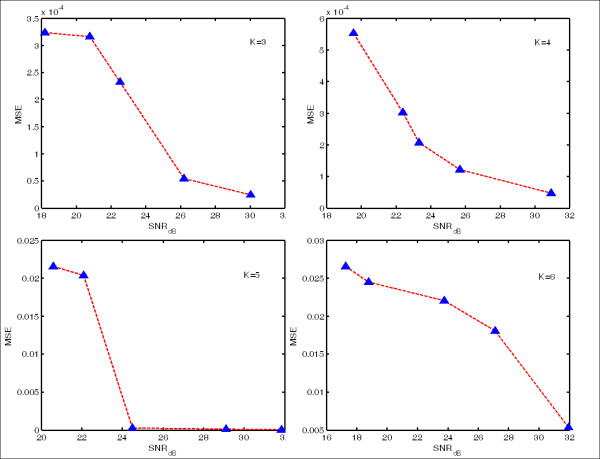
**Performance of signal reconstruction under different sparsity level and signal-to-noise ratio.** The overall mean-squared error (MSE) decreases with the increase of sparsity; for a fixed sparsity level, the MSE falls with the raise of signal-to-noise ratio (SNR).

We adopted the sparsity derivation offered by Donoho in [35]: $K=c\sqrt{m/\log\left(n\right)}$ and varied *K* by changing the coefficient *c*. By assigning *c*=1, 1.2, 1.5, 2 we accordingly get *K*=3, 4, 5, 6, after rounding each value to its nearest integer. For each *K* the simulations are repeated five times by regenerating random **x**_*o*_and **e**. We regenerate **e** by changing *σ* to get different Signal-to-Noise Ratios (SNR). The performance of signal reconstruction is assessed by the corresponding Mean-Squared-Error (MSE) under different SNR levels. The simulation results are shown in Figure 8, with each panel refers to distinct sparsity level. One can tell from the figure that (1) the performance of reconstruction improves stably with the increase of the signal sparsity and (2) for a fixed sparsity *K*, the mean-squared-error decreases consistently with the increase of the signal-to-noise ratio. Combined, this demonstrates the effectiveness of the reconstruction scheme we employ.

To summarize, the simulation results demonstrate that given *m* measurements from the wells on the plate, we can uniquely identify which gene or group of genes from a gene library with *n* (*m* ≪ *n*) genes has been knocked down. Specifically, a set of 48 siRNAs acting as group identifiers are able to infer the interfering profile of a group of 146 target genes, which means that by applying CS principles, the size of traditionally necessary siRNA library for a certain target gene group can be reduced substantially. The good performance of signal reconstruction in this numerical experiment implies that both the original signal related to the target genes and the sensing matrix designed in this work obey the prerequisites of CS theory and related biological concerns simultaneously. Hence, it is feasible and effective to apply the CS theory to the RNAi screening related experiments to save both time and cost.

## Discussion

The proposed csRNAi screening employs a combination of several group identifier siRNAs to knockdown a much larger set of genes than the size of siRNA set. In most cases, this system can largely curtail time and cost of the RNAi experiment. The philosophy of compressed sensing is to use small number of measurements to construct or reconstruct a signal of much larger size. In this study, our experiment was performed on a limited number of genes due to resource limitation because the whole genome motif search was very time consuming.The small-scale search presented in this work (200 motifs from 600 genes) consumed about 30 hours of CPU time on a HP workstation with 4 GB of RAM. If the number of genes and length of genes increases linearly, the computation time increases exponentially. Therefore, the current computational cost is the major restriction that prevents us from generalizing this method to the whole genome. However, if we have a certain group of genes of interest, such as several genes in a particular pathway and their downstream genes, we can perform an extensive search on the gene group and derive the group identifier to build the sensing matrix. The computational cost is much more affordable than that in wet labs. Actually, the total number of six hundred genes in our simulation is far larger than a common set of genes of interest that usually contains at most a couple of hundred genes. In addition, our numerical simulations consistently showed that about one third of traditionally necessary siRNAs is sufficient to identify and silence the effective genes with high precision. Therefore, by focusing on a certain gene group, the proposed system is very promising to help reduce the cost and even derive new scientific discoveries.

Another highlight is the good performance of siRNA feature extraction and prediction method proposed in this work. Although we focus on developing a new system for RNAi screening, the accuracy of siRNA prediction will significantly affect the result because 1) if the siRNAs fail to knockdown target genes, the following work is meaningless and 2) if there are no sufficient numbers of group identifier siRNAs, it is very difficult to design a network or perfectly reconstruct the signals. Therefore, we documented our proposed feature extraction scheme and siRNA prediction method quite extensively, especially in the Additional file 1.

The classification results for each feature group are listed in Additional file 1: Table S5, Additional file 1: Table S6, Additional file 1: Table S7, Additional file 1: Table S8, Additional file 1: Table S9, Additional file 1: Table S10, Additional file 1: Table S11 and Additional file 1: Table S12 individually. Additional file 1: Figure S5 illustrates the prediction accuracy with and without SVM-RFE on data set 2. Additional file 1: Figure S6 shows the contribution of each feature group to the overall feature space after feature selection within the combined features. It can be seen from these tables and figures that the average testing results trained by data set 2 generally outperform the other two training data sets. The testing results of different data sets are also very consistent if trained by data set 2. Thus, the rules used to extract position specific features (PSF) and sequence position specific features (SPSF) are derived using data set 2. We choose data set 2 to obtain the rules because it contains a large enough number of training instances and a wide enough activity value gap between positive and negative samples.

Our proposed position specific feature (PSF) is more robust than the determined rule derived from specific data, which is a major advantage of our method. Additional file 1: Figure S4 shows that our proposed position specific rules significantly outperform Reynolds’ rational siRNA design and Huesken’s artificial neuron network method. Sequence position specific features (SPSF) and Thermodynamic (TD) features are similar in that both of them employ 2-bp (base pair) length short sequence patterns to define features. Our method is also able to extract different features using different feature training data for SPSF. It specifies the pattern directly without parameterization, summarization or other kind of procedures. The PSF and SPSF offer information of desired patterns of sequences at several important positions and the maximal length of the successive positions is 2. For *N*-gram and *N*-GSK features, although the prediction accuracy is high for both, we observe some over-fitting issue from the fact that the classifier containing plenty of samples as support vectors. The position coding (PC) features are composed of features extracted from single position along with two successive positions. Although claimed with the best performance in [21], the modified PC feature in our experiment is still inferior to our proposed PSF feature. Treated as objects with similar shape to cells, the transformed gene sequences possess texture and moment features. It is suitable for a sequence with 15–25 bp length transformed to a round shape to avoid being treated as random noise and too much overlap in a matrix representation (see Additional file 1). For the first time the speech features are introduced into the siRNA signal processing, its performance is as good as PSF and SPSF features and deserves further study. The wave-like nature of the gene sequence, together with the underlying linear prediction coding mechanism, inspires us to perform the speech signal processing technique on the gene sequences. To summarize, when considered individually, the PSF and SPSF have higher prediction accuracy than other groups while the *N*-GSK bottoms the rank. The feature selection procedure improves *N*-Gram, *N*-GSK, PC, Image and Speech features dramatically when compared with other groups, and it pops the PC feature to the top of the rank.

The last issue is that we observe that a minority of simulations suffer unsatisfactory mean squared error. This implies that the compressed sensing matrix is not always ideally incoherent. Actually, for the column pairs of the sensing matrix in our experiment, 262 inner products (about 2.5%) are larger than 0.6. However, we reasonably expect that the normalized error would decrease as the dimension of the sensing matrix increases. This is because the sensing matrix would become relatively sparse and therefore more column pairs would yield small inner products.

## Conclusions

In this paper, we establish a conceptual model, compressed sensing RNAi (csRNAi) system, by applying the compressed sensing (CS) theory to the large scale RNA interference (RNAi) screening. The CS theory guarantees that under the sparsity and incoherence conditions, one can recover certain signals or images from far fewer samples or measurements than traditionally considered necessary. For the first time, we introduce this theory into large scale RNAi screening and present an example illustrating that it is possible to screen the target gene set of same size using much less wells and siRNAs than traditional methods. We start with motif search to recognize candidate group identifier siRNAs. Then we build a robust machine learning based method to identify siRNAs from the candidates. In this part, we incorporate the novel features, such as image based features and speech features into siRNA prediction. We also propose a general method which is able to extract distinct PSF for different gene sets. This method considers both desired and undesired position compositions for a single nucleotide. With these novelties, a classifier is established after SVM-RFE feature selection. This classifier substantially improves the prediction accuracy comparing with the literature. Since we have identified the group identifier siRNAs and their targeted genes, we build a network and construct the corresponding sensing matrix for the system, based on the compressed sensing theory. Then we employ convex optimization technique to reconstruct the signal which contains the information of the applied siRNAs. Simulation results solidly demonstrate this concept is easy to implement and can achieve desired results. In conclusion, a csRNAi screening system has been proposed and validated by numerical experiments. With the result, the current RNAi screening can be improved to be more efficient and less time and cost consuming.

## Competing interests

The authors declare that they have no competing interests.

## Authors’ contributions

XBZ & JF & HT conceived the original idea, wrote the main body of the manuscript and implemented experiments. JGB & JGD attended the discussion of the work and revised the manuscript. All authors have read and approved the final version of this manuscript.

## Authors’ information

Hua Tan and Jing Fan Co-First Authors.

^**†**^The program/software is available upon request.

## Supplementary Material

Additional file 1 **This file consists of two parts of Additional file **1**.** The first part gives a detailed description of the methods and results of the siRNA prediction. The second part lists the results of the numerical experiments, i.e., the selected siRNAs and associated genes. Click here for file
