# Supplementary material for: A Computational model for compressed sensing RNAi cellular screening
Source: BMC Bioinformatics. 2012 Dec 27;13:337. doi: 10.1186/1471-2105-13-337 (PMC3544734; doi:10.1186/1471-2105-13-337)
Supplement: Additional file 1 — This file consists of two parts of Additional file 1. The first part gives a detailed description of the methods and results of the siRNA prediction. The second part lists the results of the numerical experiments, i.e., the selected siRNAs and associated genes. [file 1471-2105-13-337-S1.doc]

Supplementary Materials for

**A Computational Model for Compressed Sensing RNAi Cellular Screening**

Hua Tan, Jing Fan, Jiguang Bao, Jennifer G. Dy, Xiaobo Zhou

Correspondence: [xzhou@tmhs.org](mailto:xzhou@tmhs.org)

Co-First Authors.

**Part I: siRNA Prediction**

In this part, we first describe the methods and materials we employed in this paper to predict siRNAs. Then we present results and discuss several interesting scientific issues.

**Methods and Materials**

**Data acquirement and structure**

The data used in this work is from Huesken et al. 2005 [1]. It contains 2431 siRNAs and their interference activities that indicate the effectiveness of the siRNA. For each siRNA, we use 19-bp length sequences in the Huesken’s data. The original data do not specify an activity threshold to separate 2431 siRNAs into effective and ineffective classes. Therefore, we first rank the samples with their activities from high to low and then build the data sets in our experiment described in Figure S1. Three data sets are tested in our studies, with Data Set 1 as the subset of Data Set 2 and 3, while Data Set 2 as the subset of Data Set 3. We also mark the normalized activities values on top of the color bar.


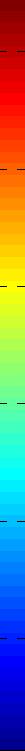


**Data Set 1: Top 200 + End 200**

**Data Set 2: Top 300 + End 300**

**Data Set 3: Top 500 + End 500**

**siRNA activity**

**High Low**

Cut off: 0.91 0.878 0.832 0.476 0.393 0.321

**Figure S1** Data structures. Squares in different color indicate different data sets with effectiveness threshold.

**Feature Extraction**

1. **Position specific features (PSF)**

Position specific features are the most important features that biologists discover by biological experiment and statistical studies. Most existing siRNA design methods are based on this category. In addition, in our problem, the motif search results present some variances of a certain motif, which means some positions are not exactly the same at all locations. Therefore, we intend to study the importance of each position to decide whether we allow such variances or not, and which will or will not affect the motif to be a candidate siRNA. However, the existing position specific features and their corresponding scoring systems are derived from various siRNA libraries. Consequently, the conclusion cannot be simply generalized to other data set. To solve this problem, we develop a method to study the position significance of any given samples statistically.

**Table S1**: The coding method for each gene

| 1 | 2 | 3 | 4 | 5 | 6 | 7 | 8 |
| --- | --- | --- | --- | --- | --- | --- | --- |
| A | no A | G | no G | T | no T | C | No C |
| 1000 | 0111 | 0100 | 1011 | 0010 | 1101 | 0001 | 1110 |
| 9 | 10 | 11 | 12 | 13 | 14 |  |  |
| K(G/T) | M(A/C) | R(A/G) | Y(C/T) | S(G/C) | W(A/T) |  |  |
| 0110 | 1001 | 1100 | 0011 | 0101 | 1010 |  |  |

We first encode the common 14 appearances of a gene position. The codes are listed in Table 1. For a given position, there are A, G, T and C four possible cases. These 14 representations are widely used in the biological community.

Then we employ the similar technique of hypothesis testing in microarray experiments. Our sample size is not large, which is usually 200 to 300 samples, so we need to perform random permutation to enlarge the testing sample size. For each permutation, we randomize the order of the instances in the sample. Given training samples, we record frequencies of the 14 events. In addition, we record frequencies of all random permutations. Our test statistics are the frequencies of the 14 event at different positions and we calculate the *p*-value as follows. For the *i*-th permutation, $i=1,\cdots,N$, we permute the feature matrix and compute the test statistics $t_{1,i},\cdots,t_{m,i}$for each hypothesis $H_{j},j=1,\cdots,m$. Then for two-side alternative hypotheses, the *p*-value for hypothesis$H_{j}$is calculated by:

$$p_{j}=\frac{\sum_{i=1}^{N} I(|t_{j,i}|\geq|t_{j}|)}{N}$$

Wheredenotes the indicator function which equals to 1 if the condition is satisfied and 0 otherwise.

Usually the position specific features or existing scoring systems of the siRNAs are derived from the positive data sets which are collections of effective siRNAs. Here we improve by adding the negative training data sets that contain ineffective siRNAs. There are two advantages to study the negative data: 1) if some instances exist in both positive and negative data set, it is considered as a nonspecific event of the given data set. This helps us exclude the situation that we consider one event is statistical significant if we only process the positive data; 2) from the statistical study on the negative data, we can derive some penalty rules which assist in prohibiting some undesired position variances in effective siRNAs.

For each position in a certain sequence, we have 14 p-values for 14 events. Usually events with p-value less than 0.05 have statistical significance. Therefore we focus on events with p-value lower than the threshold 0.05 for both positive and negative data.

We notice that there are some overlaps and complements among the 14 events. For example, the overlaps of *no A* and *K (G/T)* are *G* and *T*, and the *A* and *no A*, *K* and *M* are two pairs of complementary pairs. In addition, among 14 events at a certain position, there might be more than one event with a small p-value. For example, for the positive data and position 1, there are six events with *p*-value less than 0.05. They are no *G* (1011), *T* (0010), no *C* (1110), *K* (0110), *W* (1010) and *Y* (0011).

Now we describe how we derive the final rules as position specific features derived from the statistical analysis. We take position 1 as an example. The summation of all possible codes of each position is (3262). From the negative data analysis at position 1, the only event with *p*-value less than the threshold is *G* (0100). Then we subtract the negative code from the positive one and get (3162) which we consider as the temporary decision. Next we derive positive and negative rules from the temporary decision. Here we adopt the majority vote strategy. For positive rules, the number of voters is six and the majority wins at code position 1 and 3. We represent the win as one while the failure is represented as 0. Therefore, the positive decision becomes 1010, which indicates that A or T is desired at position 1. For the negative rules, on the other hand, we vote at position in the temporary code with code less than 0 (see the next paragraph for an example). We adopt the same encoding as the positive vote. On the positive positions we give 0 to this coding position. Therefore, the negative decision for this position is 0000, which means no penalty for this position.

Here is another example with penalty code. At position 2, the summation of positive codes is 2240 while the negative one is 0012. The numbers of positive and negative voters are 4 and 2 respectively. The temporary decision is 223-2. According to the approach described previously, the positive decision is 1110 and the negative decision is 0001. Therefore, the penalty at position 2 is on the appearance of C. In addition, we notice that the positive decision and the negative one are complementary to each other. In this case, we will delete the penalty to remove the redundancy.

Input data

Positive data Statistics

Negative data Statistics

Positive code

Negative code

Temporary decision

Majority vote

Majority vote

Positive decision rules

Negative decision rules

Subtraction

**Figure S2** Flowchart of derivation of the position specific decision rules.

Figure S2 illustrates the approach describing how we derive the rules to extract the position specific features for the input data. Table 2 lists the position specific rules derived by Data Set 2. When the mRNA content appears at the corresponding position, the feature is 1 and otherwise it is 0. The feature vector for the 19-bp length sequence ‘TAACACCATTATCTGATGC’ is 1111101001000100000. This sequence is a candidate siRNA with activity value 0.928. Our strategy statically analyzes the data which is very flexible for different data sets.

**Table S2** Position specific rules derived by Data Set 2

| **Positive Rules Derived by Data Set 2** | | | | | | | |
| --- | --- | --- | --- | --- | --- | --- | --- |
| **Position** | **1** | **2** | **4** | **5** | **6** | **7** | **9** |
| **Content** | A/U | A/U | A/G | A/G/C | C | A | C/U |
| **Code** | 1010 | 1010 | 1100 | 1101 | 0001 | 1000 | 0011 |
| **Position** | **10** | **11** | **13** | **16** | **17** | **18** | **19** |
| **Content** | A | G | A/U | A | G | C | G/C |
| **Code** | 1000 | 0100 | 1010 | 1000 | 0100 | 0001 | 0100 |
| **Penalty Rules Derived by Data Set 2** | | | | | | | |
| **Position** | **3** | **6** | **7** | **11** | **19** |  |  |
| **Content** | U | U | C | C | A |  |  |
| **Code** | 0010 | 0010 | 0001 | 0001 | 1000 |  |  |

1. **Sequence position specific features (SPSF)**

Biologists suggested that besides the position specific features, there might exist some patterns at certain consecutive positions on siRNAs [2]. These patterns are short sequences containing 2 to 3 basic pairs (bp). We follow the same manner of the statistical analysis of the position specific feature in the previous section to extract the sequence feature.

In our study, we examine the 2-bp length sequences. There are 16 possible patterns for 2-bp sequence. It can be expressed as a 2-order Hidden Markov Model (HMM) with the percentages of AGTC at first point as the initial probabilities and 4-by-4 transition matrix with elements representing frequencies of all possible 2-bp lengths instances. However, 2-order is too short for us to derive some meaningful conclusions with HMM. Instead, we modify the 2-order HMM to the format in which each element of the transition matrix representing frequencies of 16 possible patterns following the similar manner of PSF rule derivation. In this case, we have 16 events as 16 possible combinations. This case is much simpler than that in the previous part because these 16 cases have no overlaps or complementary relationships between each pair. We only record events with statistical significance for each position. Tests are performed on both positive and negative data. We delete shared patterns that occur in both positive and negative data at a certain position. The penalization is not applied to this feature group.

Table S3 summarizes the sequence position specific feature rules derived by Data Set 2. Each element in the feature vector for a sequence is 1 if the sequence at that position meets the rule and 0 otherwise. There are 18 features in this category.

**Table S3** Sequence Position Specific Feature Rules Derived by Data Set 1

| Sequence Position Specific Feature Rules Derived by Data Set 2 | | | | | | |
| --- | --- | --- | --- | --- | --- | --- |
| Position | 1,2 | 2,3 | 3,4 | 4,5 | 5,6 | 6,7 |
| Content | TA/TC | AA | AG | GG | AG | CA/CT |
| Position | 7,8 | 8,9 | 9,10 | 10,11 | 11,12 | 12,13 |
| Content | AG | N/A | TG | GG | GC/TG | TC/CA |
| Position | 13,14 | 14,15 | 15,16 | 16,17 | 17,18 | 18,19 |
| Content | AT | AA/TC | CA | AG/TG | GA | GG/TG |

**Thermodynamic Feature (TD)**

The thermodynamic feature of a sequence represents the Gibbs free energy stabilities [3]. The thermodynamics provide a foundation for predicting secondary structure and stability, both of which are related to RNA function. Parameters given by INN-HB (individual nearest-neighbor based on hydrogen bonding) model are employed to calculate thermodynamic properties. The feature space contains 21 features and 18 of them are from neighbor pairs of 19 positions. These features are based on the expression of 2 bp length sequence as well. The $\Delta G_{37}^{\circ}$ values of different combinations described in Xia, T. et al. (1998) [3] are listed in Table S4. Another feature is the terminal information which is 1 while the sequence ends with A or T and 0 otherwise. The last feature is the total energy of the sequence. We detect the 4 bp-length short hairpin structures and include this information in the total energy. The total energy is calculated as:

$$\Delta G^{\circ}(\text{duplex})=\Delta G_{\text{init}}^{\circ}+\sum_{j} n_{j}\Delta G_{j}^{\circ}(\text{NN})+m_{\text{term-AU}}\Delta G_{\text{term-AU}}^{\circ}+\Delta G_{\text{sym}}$$

$\Delta G_{\text{init}}^{\circ}$ is the free energy of initiation. Each $\Delta G_{j}^{\circ}(\text{NN})$term is the free-energy contribution of the *j-*th nearest neighbor with $n_{j}$ occurrences in the sequence. $\Delta G_{\text{term-AU}}^{\circ}$ is terminal AU pair free-energy parameter and its number of incidences is$m_{\text{term-AU}}$. And if hairpin structure exists in the sequence, the$\Delta G_{\text{sym}}$term will be added.

**Table S4** Thermodynamic parameters

| $\Delta G_{37}^{\circ}(\text{NN})$ **values in INN-HB model described in Xia, T. et al. (1998)** | | | | | | | | |
| --- | --- | --- | --- | --- | --- | --- | --- | --- |
| Expression | AA | AG | AT | AC | GA | GG | GT | GC |
| $\Delta G_{37}^{\circ}$ | -0.93 | -2.08 | -1.10 | -2.24 | -2.35 | -3.26 | -2.24 | -3.42 |
| Expression | TA | TG | TT | TC | CA | CG | CT | CC |
| $\Delta G_{37}^{\circ}$ | -1.33 | -2.11 | -0.93 | -2.35 | -2.11 | -2.36 | -2.08 | -3.26 |

1. **N-gram Feature**

An *N*-gram is a sub-sequence of *N* element of a given sequence. *N*-gram is widely used in areas such as statistical speech processing and genetic sequence analysis. *N*-gram feature denotes the occurrence of *N*-length pattern of a given alphabet. For DNA or RNA, each position of *N* length pattern has A, G, C and T/U four possible components and the number of all the possible combination is $4^{N}$. For example, while *N*=2, all the possible combinations are { AA, AG, AC, AT, GA, GG, GC, GT, CA, CG, CC, CT, TA, TG, TC, TT}, and 2-gram feature vector of sequence AAGCCGCTAA can expressed as 1100001101111000. Here we test 2, 3, 4-gram features, and this feature space contains $4^{2}+4^{3}+4^{4}=336$features. When *N* increases, the feature vector becomes more sparse because 19-bp length is too short to contain long and various patterns. For example, when *N*=4, there are 256 possible patterns. However, only 16 patterns exist in a 19 bp-length sequence. This means at least 240 entries of a 1-by-256 vector are zero. These features are designed to reveal the relationship between effectiveness and sequence patterns or motifs.

1. **N-GSK Feature**

GSK (general string kernel) is based on the mismatch string kernel (MSK). The *(N,m)-*MSK maps feature space generated by shared occurrences of fixed *N*-length subsequences which are different at most *m* mismatches. In our study, the mismatch position is set to zero. Then the extracted features are the occurrence of different *N*-length patterns. Similar to *N*-gram feature, *N*-GSK maps feature space indexed by all possible subsequences of siRNAs of fixed length. For the example in the *N*-gram feature part, AAGCCGCTAA, 2-GSK feature vector is 2100002101111000. The possible combinations and their order are the same as those in *N*-gram features.

1. **Position coding feature (PC)**

Instead of performing mathematical analysis on the sequence, position composition features are encoded sequences without any statistical analysis, counting or binaryzation. For each position, there are A, G, T and C four possible expressions. The code for A is 1000, for G is 0100, for T is 0010 and for C is 0001. Therefore, a 19-bp-length siRNA sequence is encoded by a $4\times19=76$ sequence and we employ the 1-by-76 vector as the 1-bp position composition coding features for a siRNA sequence. Similarly, for two consecutive positions, there are 16 possible patterns, and we also represent occurrence of one pattern as 1. Therefore for each consecutive 2 bp patterns, we have a 16 length code with only 1 non-zeros entry and 15 zeros. And for 19-bp-length sequence, there are 18 2-bp neighboring neighbors, thus the size of feature vector for the 2-bp position composition coding feature is$18\times16=288$. The total number of the position composition coding feature is 364.

1. **Image features**

Another feature set is image features which include wavelet feature, Haralick feature and moment feature [4]. This feature space is novel in the genetic sequence processing. If the gene sequence is represented by binary indicator sequence, this transforms a one-dimensional gene sequence into a two-dimensional image. The binary indicator sequence contains either one or zero at a given site and one denotes the corresponding character is located at the site. For example, the sequence AAGCCGCTAA (the same example sequence given in the Gram feature part) can be expressed as binary indicator matrix with rows represent A, G, T and C respectively:

$$\left[\begin{matrix} 1100000011 \\ 0010010000 \\ 0000000100 \\ 0001101000 \end{matrix}\right]\begin{matrix} A \\ G \\ T \\ C \end{matrix}$$

If treated as images, the binary indicator matrix of a siRNA has the appearance of waveforms and it looks like random noise because of the sparsity (see Figure S3). If we can transform the binary indicator matrices to other formats that may contain some shape information, it is possible to extract features such as texture and moment features. We notice that in cell image classification, especially in RNAi genome wide screen, there are several categories of phenotypes which are signified by different texture features. Inspired by the cell image processing, we would like to transform the binary indicator sequence matrix to the one with round shape similar to cells. To do this is simple. Our strategy is to slice a disk into *N* slides with equal angle, where *N* stands for the length of the sequence. Then on each spoke for the center of the disk to the edge, we put a four-character length code on it. The code for A is 1000, for G is 0100, for T is 0010 and for C is 0001, which is the same as previous part. Then we put the coded disk onto a 9-by-9 matrix centered at pixel (5, 5) and each element is a pixel. Obviously as *N* increase, a 9-by-9 matrix cannot differentiate small angles. More specifically, in the center area, several spokes may lie in a same pixel. In our case, *N* is the length of siRNAs and equal to 19. If one pixel contains multiple spokes, we take the summation of codes. Moreover, each spoke is four pixels length. A positive training sample and a negative one are illustrated in Figure S3.


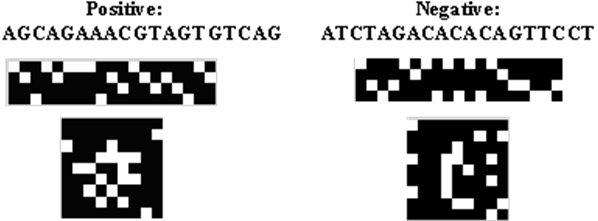


**Figure S3** Positive and negative training samples of transformed images

By representing each sequence as an image, we can employ the similar strategy as that in cell image classification to extract the features [4]. The first feature category is Haralick co-occurrence texture features which can be extracted from the gray level spatial-dependence matrices. There are totally 14 co-occurrence texture features are defined. They are Angular Second Moment, Contrast, Correlation, Sum of Squares, Inverse Difference Moment, Sum Average, Sum Variance, Sum Entropy, Entropy, Difference Variance, Difference Entropy, Information Measures of Correlation, and Maximal Correlation Coefficient. In our implementation, the first 13 is employed because the last one is always the same for all the sample sequences.

Another category is wavelet coefficients. Gabor wavelets are directly linked with Gabor filter because they can be designed for number of scales and orientations. The Gabor filters are considered as dilations and rotations tunable line detectors. In addition, the texture information can be characterized by the statistical information, such as mean and variance in a certain patch. It makes Gabor wavelet features widely used in different image analysis applications, such as image retrieval, segmentation and classification.

Here we adopt the strategy described in Manjunath, et al. 1996 [5]. With five scales and 7 orientations, we have a 70 elements feature vector for each input sample images, which is$[\mu_{00}\sigma_{00}\mu_{01}\sigma_{01}\cdots\mu_{46}\sigma_{46}]$. $\mu_{ij}$is the mean and $\sigma_{ij}$ is the standard deviation of the magnitude of the transform coefficients and *i* stands for scale while *j* refers to the orientation.

The last feature category of image features is Zernike moment features. Zernike moments are derived from Zernike polynomials that present an orthogonal set [6]. The orthogonality provides the uniqueness of features extracted by the polynomials. In addition, the moments defined in polar coordination are invariant with respect to their magnitude under rotation. In our implementation, we first locate the centroid of each image, and then define the maximum radius *R* as the farthest pixel from the centroid. Finally the images are normalized with the center of mass and the maximum radius *R*. The pixel (*x, y*) in the original image is represented by$(\rho,\theta)$, which is the projected image from a Cartesian to polar coordinate. With the notation $I(x,y)$ and $I(\rho,\theta)$ being intensities of original and projected image respectively, the Zernike moment for each image is defined as:

$$Z_{nm}=\frac{n+1}{\pi}\sum_{x'} \sum_{y'} V_{nm}^{*}(\rho,\theta)I(\rho,\theta)$$

where $0\leq m\leq n$,$(n-m)$ is even, $\rho\leq1$, $V_{nm}^{*}$ is the complex conjugate of $V_{nm}$, which is defined over the unit disk as

$$V_{nm}(\rho,\theta)=R_{nm}(\rho)\exp(jm\theta)$$

where$R_{nm}(\rho)$is the Zernike polynomials given by

The magnitude of $Z_{nm}$will be used as feature. We choose *n*=12 to obtain 49 moment features in all. To summarize, we get 132 image features totally in this part.

1. **Speech features**

In speech signal processing and recognition, the human voice is converted to machine-readable signals, for example, to the binary code from a string comprised from a certain alphabet [7]. Similarly, genomic sequences are comprised by an alphabet of four characters, namely A, G, T/U and C. The speech recognition has been studied for a long time and there are several effective methods to process the speech signal. Based on the similarity between speech sequences and DNA or RNA sequence, we employ the similar feature extraction strategies for siRNA as that in speech. Many different feature types are used in various applications of speech processing. Linear prediction coefficients (LPC), mel-frequency cepstrum coefficients and wavelet features have been found to be the most effective features for speech recognition.

To digitalize the alphabet representation of siRNAs, we adopt the approach as mentioned in previous part. A 19 bp-length siRNA sequence is represented by a $19\times4=76$ bp-length sequence. It is identical to the 1-bp position composition coding (PC) features.

We first analyze the LPC feature. Linear predictive analysis of speech signals has become the predominant technique for estimating basic parameters of speech. It is based on the auto-regression (AR) model to predict a set of parameters or predictor coefficient that minimizes the mean square errors between the actual signal and the prediction. The estimate signal at time *n* can be described as:

$$\hat{s}(n)=\sum_{k=1}^{p} \alpha_{k}s(n-p)$$

where *p* is the order of prediction, $\alpha_{k}$is the prediction coefficient and$s(n)$is the signal. There are *p* coefficients $\alpha_{k}$ used as *p*-dimensional vectors to represent a short sequence, which is a speech frame in speech recognition and a siRNA sequence in our problem. The prediction coefficients $\alpha_{k}$ can be determined by solving the optimization problem that minimizes the mean square error (MSE) between the real signal and its estimation:

$$\{\alpha_{k}\}=\mathop{\mathrm{argmin}\sum_{n=1}^{N} (s(n)-\hat{s}(n))^{2}}\limits_{\alpha_{k}}$$

where *N* is the number of observation samples. By employing order 8, which is the length of two base pair expressions with coding, we have 8 coefficients as the LPC features.

Although widely used in speech recognition, LPC is limited due to linear prediction nature. Mel-frequency cepstrum coefficient (MFCC) is one of the most popular and successful coefficients that represent audio signals. MFCC is widely applied in speech/speaker recognition and increasingly in music information retrieval applications. We intend to explore the application of these coefficients in the audiowave-like siRNA expressions. As in LPC calculation, the siRNA expressions are treated as an audio sequence. For a digitalized signal $S=\{s_{n}\},n=0,1,2,\cdots,N-1$, the standard derivation of MFCCs is stated as follows:

1) Pre-emphasis: A first order difference equation is employed to pre-emphasis the signal.

$$s_{n}^{'}=s_{n}-\lambda s_{n-1}$$

where $\lambda$ is a pre-emphasis factor and $0\leq k<1$, and usually it is set to 0.97. When the sequence is long, windows are typically applied to divide the sequence into frames. To alleviate the effect of discontinuity at the window boundaries, a Hamming window is the common choice:

$$s_{n}^{''}=s_{n}^{'}[0.5384-0.4616\cos(\frac{2\pi(n)}{N})],n=0,1,2,\cdots,N-1$$

2) Frequency analysis: Discrete Fourier transform (DFT) transfers the time domain signal $s_{n}^{'}$ into the frequency domain by:

$$f_{k}=|\sum_{n=0}^{N-1} s_{n}^{''}\exp(-2\pi nki/N)|$$

where *k* is in the range $[f_{L},f_{H}]$ , $f_{L}$ and $f_{H}$ are lower cut-off and upper cut-offer frequency respectively. Here we take the magnitude of the transformed values and calculate the short term power spectrum$P(f)$.

3) Spectral Warping: The frequency representation$P(f)$ is warped along its frequency axis *f* into mel-frequency axis using:

$$Mel(f)=2595\log_{10}(1+\frac{f}{700})$$

The parameters are chosen to approximately reflect the human’s ear perception. Then we convert it to log-domain by:

$$m_{k}=\log(Mel(f_{k}))$$

Then the warped power spectrum is convolved with a set of triangular band pass filter banks. The output of filter banks is represented as $\theta(m_{k})$and *m* denotes the mel-frequency axis and *k* is the index of filters and totally *K* filters. These filters are equally spaced along the mel-frequency axis. Then *K* outputs$\alpha_{k}=\ln(\theta(m_{k})),k=0,1,2,\cdots,K-1$are acquired.

4) Orthogonalization: In order to eliminate the correlation among *K* mel-scaled log filter bank outputs, we apply the discrete cosine transform (DCT) after the spectral warping and derive the coefficients as:

$$c_{n}=\sum_{k=0}^{K-1} \alpha_{k}\cos\left(\frac{\pi n}{K}(k-0.5)\right),n=0,1,2,\cdots,N-1$$

5) Liftering: Liftering, which is a re-scale approach, is applied to solve the problem, in which the higher order cepstrum of MFCCs are numerically small, which results in a wide range of variance of cepstral coefficients. This process can be summarized as:

$$c_{n}^{'}=(1+\frac{L}{2}\sin\frac{\pi n}{L})c_{n}$$

where *L* is the liftering value.

The similarity between audio waves and DNA or RNA sequence makes it possible to treat DNA or RNA sequences as audio waves. We will do some parameter optimization for DNA sequence with MFCC in our future work.

Researchers also take psychophysics of hearing into consideration of linear prediction (LP) method, which is called perceptual linear predictive (PLP) analysis. They improve the LP method by adding the critical-band spectral resolution, the equal-loudness curve, and the intensity-loudness power law, then approximating by an autoregressive all-pole model. In addition, the PLP method is further modified by replacing the conventional critical-band short term spectrum in LP with a spectral estimate derived by filtering each frequency channel with a band-pass filter with a sharp spectral zero at the zero frequency. This procedure, which is called spectral PLP (RASTA-PLP), suppresses the slowly varying component in each frequency channel and enables the estimation to be less sensitive to slow variation noise in the short-term spectrum. The RASTA-PLP technique for each frame, or each segment of the sequence, is described as follows: 1) calculation of critical-band power spectrum: computer the spectrum as in PLP; 2) compressing transformation: transform the amplitude of spectrum with a compressing static nonlinear transformation; 3) filtering: each transformed spectral component is filtered by band-pass filter. The high-pass part alleviates the effect of convolution noise while the low-pass portion helps smooth some fast frame-to-frame spectral changes in the short-term spectral estimate. A common choice for the filter is: $H(z)=z^{4}\frac{0.2+0.1z^{-1}-0.1z^{-3}-0.2z^{-4}}{1-0.94z^{-1}}$; 4) expanding transformation: transform the filtered spectral component with an expanding static nonlinear transformation; 5) modulation: multiply the spectrum by the equal loudness curve as in PLP; 6) All-pole modeling: compute the all-pole model of the resulting spectrum.

To summarize, the reasons why we apply the speech feature in the siRNA sequence analysis are: 1) the wave-like shape similarity between audio signal and siRNA, more generally, DNAs or RNAs; 2) If we digitalize gene sequences by some coding methods, we can consider them as signals and process them with signal processing tools, especially in the transformed domain; 3) Specifically, speech recognition techniques such as LPC and PLP features are employed because the linear prediction property may also existed in some positions of gene sequences. On the other hand, the speech feature extraction methods are highly correlated to human hearing systems, such as some cut-off threshold and frequency parameters. However, there are no biological or bioinformatics studies on how to generate the optimal frequency parameters to process the gene sequences using the speech recognition techniques. In our study, we follow the parameter settings in speech recognition because we vary the parameter and get similar cross validation results. In our implementation, the total number of this feature group is 199, as listed in Table S12. The first nine features are LPC with order 8. The next two groups are cepstrum coefficients and power spectrums with the same length as the coded siRNAs. Then we record 20 MFCCs with the approach described above. Then there are two groups of RASTA features. One is RASTA-PLP coefficients with order 9 and there other is RASTA-PLP cepstra.

**Results**

**Results of individual feature class**

The classification results for each feature group are listed in Table S5-S12. In all these tables, the first rows name the feature group and present the number of features in each group. The second rows present the cross validation result for each data set. The remaining rows are divided into three blocks and each part is trained by the data set listed in the second row of each block. In these three blocks, we show testing results in percentage of different data set and different SVM kernel functions.

**Table S5 SVM prediction results with cross validation on PSF**

| Position specific rules derived by Data Set 2, 20 features | | | | | | | | | | | |
| --- | --- | --- | --- | --- | --- | --- | --- | --- | --- | --- | --- |
| Data Set 1: 61.25 % | | | | Data Set 2: 67% | | | | Data Set 3: 71.3 % | | | |
|  | Data Set 1 | Data Set 2 | Data Set 3 |  | Data Set 1 | Data Set 2 | Data Set 3 |  | Data Set 1 | Data Set 2 | Data Set 3 |
| RBF | 67.25 | 68.5 | 67.1 | RBF | 72.75 | 77.5 | 73.5 | RBF | 60.25 | 66 | 70.6 |
| Poly | 77.5 | 73.83 | 68.8 | Poly | 76.75 | 80.83 | 75.6 | Poly | 65.5 | 71.5 | 75.6 |
| Linear | 65 | 67.83 | 66.8 | Linear | 62.25 | 67.17 | 67.9 | Linear | 61.25 | 66.5 | 68.8 |

**Table S6 SVM prediction results with cross validation on SPSF**

| Sequence position specific rules derived by Data Set 2, 16 features | | | | | | | | | | | |
| --- | --- | --- | --- | --- | --- | --- | --- | --- | --- | --- | --- |
| Data Set 1:64.5 % | | | | Data Set 2: 68.67 % | | | | Data Set 3:71.3 % | | | |
|  | Data Set 1 | Data Set 2 | Data Set 3 |  | Data Set 1 | Data Set 2 | Data Set 3 |  | Data Set 1 | Data Set 2 | Data Set 3 |
| RBF | 79.75 | 75.5 | 68.3 | RBF | 71.75 | 75.5 | 73.3 | RBF | 63 | 68 | 72.4 |
| Poly | 79 | 75.67 | 67.6 | Poly | 62.75 | 64 | 62.7 | Poly | 60.75 | 62.83 | 63.8 |
| Linear | 65 | 66.67 | 65.1 | Linear | 62 | 66.83 | 70.4 | Linear | 60.75 | 65.33 | 70.3 |

**Table S7 SVM prediction results with cross validation on thermodynamic features**

| Thermodynamic Features, 21 features | | | | | | | | | | | |
| --- | --- | --- | --- | --- | --- | --- | --- | --- | --- | --- | --- |
| Data Set 1: 59.5 % | | | | Data Set 2: 65.67% | | | | Data Set 3: 71.6% | | | |
|  | Data Set 1 | Data Set 2 | Data Set 3 |  | Data Set 1 | Data Set 2 | Data Set 3 |  | Data Set 1 | Data Set 2 | Data Set 3 |
| RBF | 59.75 | 65.83 | 69.4 | RBF | 59 | 65.67 | 68.3 | RBF | 62.25 | 69 | 74.3 |
| Poly | 68.25 | 72.33 | 73.2 | Poly | 62 | 68.5 | 72.2 | Poly | 59.75 | 67.83 | 72.5 |
| Linear | 60.25 | 66 | 68.3 | Linear | 59 | 65.67 | 68.3 | Linear | 59 | 65.67 | 68.3 |

**Table S8 SVM prediction results with cross validation on 2-4 Gram features**

| 2-4 Gram Features, 336 features | | | | | | | | | | | |
| --- | --- | --- | --- | --- | --- | --- | --- | --- | --- | --- | --- |
| Data Set 1: 61.5 % | | | | Data Set 2: 63.5% | | | | Data Set 3: 64.5% | | | |
|  | Data Set 1 | Data Set 2 | Data Set 3 |  | Data Set 1 | Data Set 2 | Data Set 3 |  | Data Set 1 | Data Set 2 | Data Set 3 |
| RBF | 100 | 88 | 76.1 | RBF | 100 | 100 | 85 | RBF | 100 | 100 | 100 |
| Poly | 100 | 86.17 | 75.3 | Poly | 100 | 100 | 86.4 | Poly | 100 | 100 | 100 |
| Linear | 100 | 84.5 | 73.4 | Linear | 94.75 | 95.17 | 81.6 | Linear | 86.5 | 87.83 | 89.6 |

**Table S9** SVM prediction results with cross validation on 2-4 GSK features

| 2-4 GSK Features, 336 features | | | | | | | | | | | |
| --- | --- | --- | --- | --- | --- | --- | --- | --- | --- | --- | --- |
| Data Set 1:56.25 % | | | | Data Set 2: 55.83% | | | | Data Set 3:66.3 % | | | |
|  | Data Set 1 | Data Set 2 | Data Set 3 |  | Data Set 1 | Data Set 2 | Data Set 3 |  | Data Set 1 | Data Set 2 | Data Set 3 |
| RBF | 75.5 | 70.5 | 65.8 | RBF | 92.25 | 93.33 | 80.6 | RBF | 80 | 82.67 | 84.5 |
| Poly | 98.25 | 82.5 | 71.8 | Poly | 100 | 99.83 | 85.3 | Poly | 77.5 | 80.17 | 82.6 |
| Linear | 81.75 | 74.17 | 69.2 | Linear | 68.5 | 71.17 | 67.6 | Linear | 78.25 | 80.67 | 82.3 |

**Table S10** SVM prediction results with cross validation on PC features

| Position Composition Features, 364 features | | | | | | | | | | | |
| --- | --- | --- | --- | --- | --- | --- | --- | --- | --- | --- | --- |
| Data Set 1:57 % | | | | Data Set 2: 66.17% | | | | Data Set 3:71 % | | | |
|  | Data Set 1 | Data Set 2 | Data Set 3 |  | Data Set 1 | Data Set 2 | Data Set 3 |  | Data Set 1 | Data Set 2 | Data Set 3 |
| RBF | 75.75 | 73.83 | 70.5 | RBF | 81.5 | 83 | 77 | RBF | 62.75 | 70.17 | 75.8 |
| Poly | 80.75 | 76 | 71.9 | Poly | 70.5 | 76.33 | 74.7 | Poly | 64.5 | 70.33 | 76.3 |
| Linear | 85.5 | 79.33 | 72.2 | Linear | 89.5 | 90 | 80.1 | Linear | 78.75 | 81 | 84.1 |

**Table S11 SVM prediction results with cross validation on image features**

| Image Features, 132 features | | | | | | | | | | | |
| --- | --- | --- | --- | --- | --- | --- | --- | --- | --- | --- | --- |
| Data Set 1:58 % | | | | Data Set 2: 56.83% | | | | Data Set 3:58.7 % | | | |
|  | Data Set 1 | Data Set 2 | Data Set 3 |  | Data Set 1 | Data Set 2 | Data Set 3 |  | Data Set 1 | Data Set 2 | Data Set 3 |
| RBF | 69 | 64.33 | 60.6 | RBF | 75.75 | 76.33 | 68 | RBF | 83 | 84.83 | 84.2 |
| Poly | 53 | 50.83 | 50.8 | Poly | 62.25 | 63.17 | 58.5 | Poly | 90.75 | 92.17 | 92.1 |
| Linear | 72.5 | 64.83 | 60.2 | Linear | 63.5 | 65.17 | 60.3 | Linear | 65 | 65.17 | 63.8 |

**Table S12** SVM prediction results with cross validation on speech features

| Speech Features, 199 features | | | | | | | | | | | |
| --- | --- | --- | --- | --- | --- | --- | --- | --- | --- | --- | --- |
| Data Set 1:57 % | | | | Data Set 2: 61.33% | | | | Data Set 3:64.1 % | | | |
|  | Data Set 1 | Data Set 2 | Data Set 3 |  | Data Set 1 | Data Set 2 | Data Set 3 |  | Data Set 1 | Data Set 2 | Data Set 3 |
| RBF | 100 | 83 | 72.6 | RBF | 78.5 | 80.33 | 73.5 | RBF | 64.5 | 70 | 72.1 |
| Poly | 100 | 86.83 | 75.7 | Poly | 64.75 | 66.33 | 62 | Poly | 55.5 | 57.33 | 58.1 |
| Linear | 69.5 | 67.5 | 64.5 | Linear | 70.75 | 73.67 | 69.8 | Linear | 66.25 | 70.33 | 73.4 |

For different kernel functions, RBF performs superior than other two kernels in half of the feature groups, which include SPSF, N-Gram, Image and Speech features. And polynomial kernel outperforms others in PSF, Thermodynamic (TD) and N-GSK feature groups. For the position composition (PC) feature, the simple linear kernel has higher accuracy than the other two.

Generally speaking, the average testing results trained by Data Set 2 outperform testing results trained by other two sets. We notice that all the feature groups except the image feature, the cross validation results listed in the second row of Table S5-S12 are increase accompany with the expansion of sizes of the data sets. However, for testing results, there is no such trend to indict that the larger size of the training sets, the better testing results. This suggests that when more instances are included, the testing results may even worse, since the newly included samples may not be well separable due to the nature of the date set. We notice that when Data Set 3 is used to train the classifier, the proportions of support vectors are larger than the other two data sets. This explains why Data Set 3 has relatively higher CV results but lower testing accuracies. In addition, testing results for 3 data sets are more consistent if trained by Data Set 2. Therefore, based on the above reasons, we choose Data Set 2 in the following training in this work of this data.

The rules used to extract position specific features (PSF) and sequence position specific features (SPSF) are derived from Data Set 2, which contains the top ranked 300 siRNAs and bottom ranked 300 siRNAs. We choose this data set because it contains a large enough number of training instances and a wide enough activity value gap between positive and negative samples.

**Table S13** Comparison of position specific rules

| **Gene Position** | 1 | 2 | 3 | 4 | 5 | 6 | 7 | 8 | 9 | 10 | 11 | 12 | 13 | 14 | 15 | 16 | 17 | 18 | 19 |
| --- | --- | --- | --- | --- | --- | --- | --- | --- | --- | --- | --- | --- | --- | --- | --- | --- | --- | --- | --- |
| **Reynolds’s**  **Rule^a^** |  |  | **A** |  |  |  |  |  |  | **U** |  |  | **G** |  | Three or more **A/U**  **A** at 19 and No **G/C** | | | | |
| **Huesken’s**  **Rule** | **AU** | **U** |  |  |  |  | **U**  **C** |  |  | **A** | **U**  **C** |  |  |  |  |  |  |  | **C**  **A** |
| **Ours** | **AU** | **AU** | **U** | **AG** | **AGC** | **C**  **U** | **A**  **C** |  | **CU** | **A** | **G**  **C** |  | **AU** |  |  | **A** | **G** | **C** | **GC**  **A** |

^a^Red is desired content and blue is undesired content.

The Reynolds’ rules are widely used in rational siRNA designs. They contained 6 position specific rules which were derived from a data set containing 180 siRNAs. The Huesken’s rules are derived from the top 200 and bottom 200 active siRNAs with artificial neuron network (ANN). Since we use the data set from Huesken, our conclusion is similar to Huesken’s rules. Our rules are slightly different from Huesken’s at position 7 and 11. In addition, some new rules are found in our experiment. Table S13 lists the rules defined by three different groups including ours. Figure S4 compares the classification results using features derived from these three rules. We can tell features extracted according to our rules perform much better than Reynold’s rule and slightly better than Huesken’s. By adding some new rules as features, our method improves the cross validation results. And these rules can be easily derived from different training data, which means the decision rules may vary from data to data. Therefore, the robustness of PSF extraction strategy is much better than the fixed feature or decision rule derived from some specific data which may be ineffective in other experiment situations.


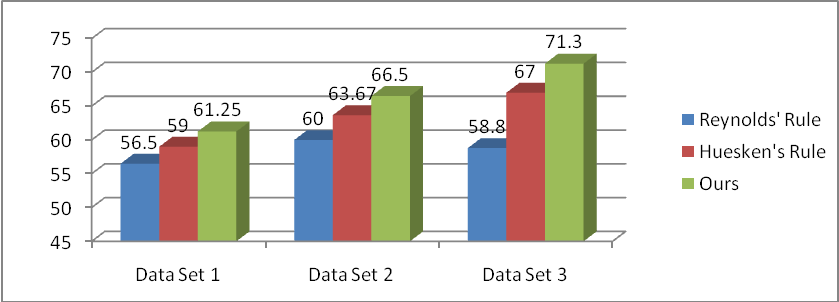


**Figure S4** Classification results of different position specific rules

Sequence position specific features (SPSF) and Thermodynamic (TD) features are similar in that both employ 2-bp length short sequence patterns to define features. However, SPSF is studied from statistical significance angle while the other is from chemical energy angle. It is reported that in Peek, 2007 [8], the thermodynamic features are well performed and ranked only second to the ‘position specific base composition features’ which are the first 76 features in our position composition (PC) features. In our SVM classification experiment, this feature group is inferior to the SPSF group. The SPSF searches for the dominant patterns of two successive positions and features are indicators of occurrence of desired patterns. The same as PSF, we are also able to extract different features using different training data. Compared with thermodynamic features, the SPSF does not contain overall information of the whole sequences, such as the overall Gibbs free energy about the siRNA sequence. Instead, it specifies the pattern directly without parameterization, summarization or other kind of transformations.

The *N*-gram and *N*-GSK features are able to extract information of longer sequence features. There are either binary occurrence indicators or counters of patterns of fixed length. Furthermore, there is no statistical or signal processing performed on them. However, if taking a close look at *N*-Gram features, classifier performs similarly to two one-class classifiers which include the majority of training samples and discard a tiny part of them according to margin values. It accurately classifies two classes with plenty of them as support vectors. When the size of the data set becomes large, testing results are better than the one trained by smaller ones.

We consider siRNAs as sequences and extract the position composition (PC) features without statistical processing. It was named as ‘*position specific base composition’* and considered as position specific features in Peek, 2007 [8]. We combine the features extracted by single position and two successive positions in our study. One can imagine how sparse it is because of the coding methods described. Claimed as the winner of the best performance in Peek, 2007, the modified PC feature in our experiment performs well but not the best. It is still inferior to PSF extracted by proposed method. If we only take the features extracted by single position as in Peek, 2007, the result is worse than our combined features.

For the Image feature of the siRNA sequence proposed in this paper, the cross validation results we achieve are not as high as others. Treated as objectives with similar shape to cells, the transformed gene sequences also possess texture and moment features. It is suitable for a sequence with 15-25 bp length to be transformed to a round shape to avoid being treated as random noise and too much overlap in a matrix representation. The testing results of this feature group are acceptable and feature selection procedure helps improve the classification results, which will be discussed shortly.

For the first time the speech features are applied in the siRNA signal processing, the performance of these features is also encouraging. When gene sequences with given alphabet are digitalized, they are considered as digital signals and processed with digital signal processing method, which is called genomic signal processing. The wave-like signature of gene sequences, together with the possible linear prediction coding mechanisms inspires us to perform the speech signal processing technique on siRNA sequences in our study. The results of this feature group are comparable to other feature groups we derived. They are superior to *N*-GSK features in the cross validation results. The testing results are also comparable to other feature groups when the classifiers are trained by Data Set 2.

**Results with feature selection on individual feature groups**

Since some feature groups have very large number of features, which may cause over-fitting problem, feature selection is very helpful and effective, not only to solve the over fitting problem but also to speed up the learning process as well as improve the model interpretability.

SVM-RFE is an effective feature selection method that was originally proposed in the gene selection. We perform SVM-RFE on each feature group to select an optimal subset of features. The results are illustrated in Figure S5, comparing classification results with and without feature selection using the same parameter setting of SVM classifier. We employ Data Set 2 to train the classifier in both cases.

**Figure S5** Classification results with and without feature selection.

From Figure S5, we can tell that for the feature groups with large number of features, such as *N*-Gram and *N*-GSK, SVM-RFE helps the classification results improve more than the ones with small feature numbers. For PSF, SPSF and thermodynamic features, the number of feature are 20, 16 and 21 respectively. SVM-RFE fails to improve the classification in SPSF and TD feature and only introduces tiny improvement to PSF by selecting 18 out of 20 features. On the other hand, the other five groups, of which the smallest feature group contains 132 features, improve at least by 6.17% among five groups. We can claim that the PC feature and *N*-Gram feature outperform the PSF and SPSF by employing the feature selection, both of which are position specific features. The numbers of features before and after feature selection are listed in Table 14.

**Table S14** Number of feature with and without feature selection performed individually.

| Feature Numbers of Different Groups | PSF | SPSF | TD | N-Gram | N-GSK | PC | Image | Speech |
| --- | --- | --- | --- | --- | --- | --- | --- | --- |
| Without Feature Selection | 20 | 16 | 21 | 336 | 336 | 364 | 132 | 199 |
| With Feature Selection | 18 | 16 | 21 | 55 | 30 | 49 | 34 | 75 |

**Results of combined feature groups**

Totally we have 1424 features if combining entire feature groups together. The cross validation results are listed in Table S15. It is clear that no obvious improvement by combining all feature groups together to train the classifier. Then we perform feature selection on the whole feature space of Data Set 2 to choose the best performed feature subspace. After the feature selection, the total number of features reduces from 1424 to 143, nearly 10% size of the original data space. The cross validation result can reach as high as 88%, which improves by 21.5% by discarding about one thousand features.

Table S15 lists the number of features that comprise the selected feature space of each feature group. For PSF, SPSF, N-Gram and PC, the proportion of each group is larger than 10%. The ratio is defined as the proportion of the total number of features of selected feature space to the number of features in the same unselected feature group. These proportions show the relative importance of the feature groups. For example, although the PSF has merely 20 features, 30% of them are kept after the feature selection on combined feature group, which is three times of the average ratio 10%. On the other hand, for TD feature and *N*-GSK features, their ratios are low and their individual performances are relatively poor comparing with other groups. The ratio is related, to some extent, to the individual feature group strength. Figure S6 illustrate numbers of features in unselected, selected individually and selected from combined feature space of each feature category.

**Table S15** Number of features with and without feature selection combined together

| Feature Number | PSF | SPSF | TD | N-Gram | N-GSK | PC | Image | Speech |
| --- | --- | --- | --- | --- | --- | --- | --- | --- |
| Without Feature Selection | 20 | 16 | 21 | 336 | 336 | 364 | 132 | 199 |
| Feature selected from combined feature space | 6 | 4 | 1 | 56 | 13 | 39 | 8 | 16 |
| Ratio | 30% | 25% | 4.8% | 16.7% | 3.9% | 10.7% | 6.1% | 8.0% |


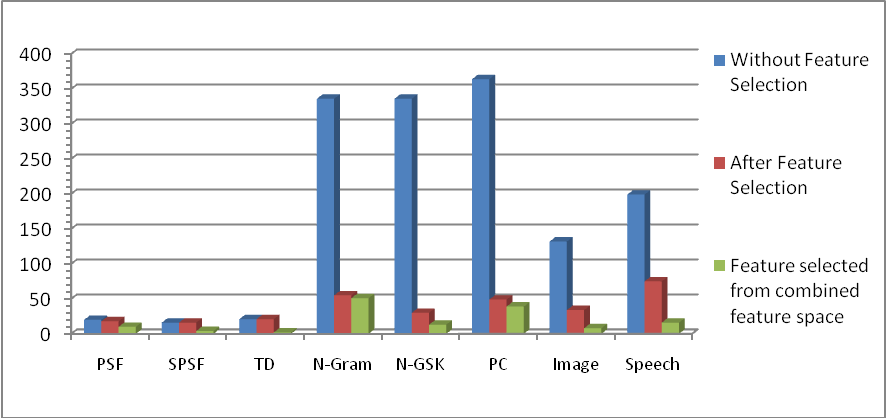


**Figure S6** Number of features with and without feature selection combined together.

**Discussion**

**Rank the features**

For each individual feature group, we rank them by their cross validation results. The rank from high to low is SPSF, PSF, PC, TD, N-Gram, Speech, Image and N-GSK. The top ranked 4 feature groups are position specified features. SPSF and PSF are the features which reveal the significant contents at several positions of siRNA sequences. PC features, which are actually a coding scheme, describe the position content of the gene sequence. And TD features are derived using some of the 2-bp neighboring information, which is similar to SPSF. The other 4 feature groups have no information about the position significance.

After performing feature selection with SVM-RFE individually on each feature group, the rank of changes to PC, N-Gram, SPSF, Speech, PSF, TD, N-GSK and Image from high to low. Due to the large feature space, which offers enough choices for feature selection, PC and *N*-Gram features pop up in the rank by keeping a 10% subset of their original size. PSF and SPSF are surpassed in the rank because of lack of improvement with feature selection due to feature space size.

**The relationship between position specific feature and position composition feature**

PSF is derived from the statistical significance studies of the original sequence data. This feature group represents the majority content at a given siRNA position considering both of positive and negative training data. It is sort of processed features because we apply statistical analysis on the data. On the other hand, the PC features are encoded or digitalized sequences of data representing by a certain alphabet. Compared with the PSF features, they are unprocessed features. In addition, PSF is not a subset of the unprocessed PC features because PSF is the result of statistical analysis instead of filtering the PC features. In Peek, 2007, the PC features were called as position specific base composition features, which were claimed as position specific features. They were considered as position specific features in Peek, 2007 because the authors performed further studies on the feature and extract position significance information from the features. In other words, the authors applied statistical analysis on the features after the features were extracted. In our studies, we consider both analyzed and unprocessed information together and classification results are better.

**Parameter selection in speech feature extraction**

In speech and speaker recognition, features are extracted with parameters selected according to human hearing perception. There are no existing studies that state the optimal parameter setting for siRNA sequence or gene sequence processed with speech processing tools. In addition, current literatures do not establish a close relationship between the frequency setting and gene sequence feature extraction and their corresponding classification results. In our studies, we employ the common cut-off frequency to extract speech features of the gene sequences. And the sampling rate is higher than common settings in speech processing because our sequence length is not as large as that of speech signals. We also compare classification results with different parameter settings and find that there are no obvious differences among distinct cut-off frequencies and sampling rates for gene sequences. The results are listed in Table S16.

**Table S16** Classification results of different parameter selection in speech features

|  | Data 1 | Data 2 | Data 3 |
| --- | --- | --- | --- |
| Cut-off frequency: 4000  sampling rate: 32000 | 57% | 61.33% | 64.1% |
| Cut-off frequency: 1000  sampling rate: 8000 | 56.5% | 60.17% | 64.2% |
| Cut-off frequency: 8000 sampling rate: 96000 | 58% | 58.17% | 64.5 |

**Part II: List of selected siRNAs and target genes**

**Table S17 A summary of 48 selected siRNAs involved in the network example in *Results* section**

| No.^a^ | Sequence | No. | Sequence |
| --- | --- | --- | --- |
| s4 | UUCUCCUGCCUCAGCCUCC | s142 | UCCAGUUUUUAAAUCCUGA |
| s23 | AGUAGCUGGGACUACAGGC | s144 | UAAUCGACUUCUUCCAUGC |
| s28 | UUUUUUUUUUUUUUUUUCA | s145 | UUCUUCUUCUUUUCCUCCU |
| s30 | UUUUUUUUUUUUUUUUGUA | s146 | ACCUCAAGUGAUCUGCCUG |
| s31 | UUUUUUUUUUUUUUUUCUG | s147 | CAGCUUUCUGAUCAAUCUU |
| s32 | UUUUUUUUUUUUUUUUAUG | s148 | UGGGAGCACAGGCCUGAGC |
| s33 | UUUUUUUUUUUUUUUUCUC | s151 | UUUUUUUUUUUUUUUUAAA |
| s42 | CCCUGCUGCCGCUGCAUCU | s152 | UUGUAUUUUUGGUAGAGAU |
| s50 | AAAAAAAAAAAAAAUAAAA | s153 | UCUGGAAUCUCAUAUUUCU |
| s53 | CUGUGACAAAUUUUUGGUC | s154 | AUAAAACAAUUAGAACACC |
| s62 | UAGCCUUCUGAGCUUUCUG | s156 | AUAGAAAAAGUAAAGAAAA |
| s70 | AAACAAAAAAAAAAAAAAA | s170 | CUCCUCGUCCUGCAGCUUC |
| s73 | UUUUUUUUUUUUUUUUGUC | s171 | UUCUGGAGUCAAUUCCUUG |
| s79 | ACAAAAUAAAGAAAAAAAA | s172 | UGGGAACUUCCUUACAGAG |
| s80 | UAAAAACUAAUAACUUAAA | s181 | GGUAGCUUUGUCAAACAAG |
| s85 | UCAAAAUCCUGGGCAACAU | s183 | UCGAUUUUGGUUGAAGUGG |
| s124 | AAAAAAAAAAAAAGAAAAG | s186 | AGCUCAGGACUUUGAGACC |
| s125 | CUAUUAAAAAAACAAAAAU | s187 | CUUUACUAAGGAGCUCCUG |
| s129 | CAACUAUGAACUCCUAACC | s189 | AAAUAUAUAUAUAUAUAUA |
| s130 | AUUAAAAAAAUUUUGCAUU | s191 | UUGUCUGCCAUUUUCUUAA |
| s131 | AAAACAACACACAACAUAC | s193 | UCACGCCUGUAAUCCCAGC |
| s132 | UCAUUCUGAAUGUCCGCCA | s194 | CAUCAGGAAGAUGUUUUGG |
| s134 | UUAUUCAACUGGUCUCAAG | s199 | UGGCGAUUUCCACCAUCUC |
| s135 | UCAAUGUAGGUGCCCUCAA | s200 | UGCCUUAUCAUUCUUGUCG |

^a^The item ‘No.’ refers to its index in this work, and ‘Sequence’ represents the nucleotide motif of 19bp. See the text.

**Table S18 A summary of 146 target genes involved in the network example in *Results* section**

| No.^a^ | Gene ID | No. | Gene ID | No. | Gene ID | No. | Gene ID |
| --- | --- | --- | --- | --- | --- | --- | --- |
| g501 | Hs#S4323237 | g580 | Hs#S4323316 | g697 | Hs#S4323433 | g843 | Hs#S4323894 |
| g502 | Hs#S4323238 | g586 | Hs#S4323322 | g699 | Hs#S4323435 | g847 | Hs#S4323898 |
| g503 | Hs#S4323239 | g590 | Hs#S4323326 | g702 | Hs#S4323438 | g854 | Hs#S4323905 |
| g504 | Hs#S4323240 | g593 | Hs#S4323329 | g705 | Hs#S4323441 | g857 | Hs#S4323908 |
| g505 | Hs#S4323241 | g594 | Hs#S4323330 | g708 | Hs#S4323444 | g859 | Hs#S4323910 |
| g506 | Hs#S4323242 | g595 | Hs#S4323331 | g714 | Hs#S4323450 | g862 | Hs#S4323913 |
| g508 | Hs#S4323244 | g598 | Hs#S4323334 | g715 | Hs#S4323451 | g869 | Hs#S4323920 |
| g510 | Hs#S4323246 | g599 | Hs#S4323335 | g722 | Hs#S4323458 | g877 | Hs#S4323928 |
| g514 | Hs#S4323250 | g600 | Hs#S4323336 | g725 | Hs#S4323461 | g886 | Hs#S4323937 |
| g516 | Hs#S4323252 | g601 | Hs#S4323337 | g727 | Hs#S4323463 | g891 | Hs#S4323942 |
| g517 | Hs#S4323253 | g604 | Hs#S4323340 | g735 | Hs#S4323471 | g901 | Hs#S4323952 |
| g518 | Hs#S4323254 | g606 | Hs#S4323342 | g747 | Hs#S4323483 | g905 | Hs#S4323956 |
| g521 | Hs#S4323257 | g607 | Hs#S4323343 | g748 | Hs#S4323484 | g909 | Hs#S4323960 |
| g522 | Hs#S4323258 | g614 | Hs#S4323350 | g755 | Hs#S4323491 | g919 | Hs#S4323970 |
| g523 | Hs#S4323259 | g616 | Hs#S4323352 | g757 | Hs#S4323493 | g923 | Hs#S4323974 |
| g524 | Hs#S4323260 | g617 | Hs#S4323353 | g763 | Hs#S4323499 | g944 | Hs#S4323995 |
| g525 | Hs#S4323261 | g618 | Hs#S4323354 | g764 | Hs#S4323500 | g956 | Hs#S4324007 |
| g526 | Hs#S4323262 | g619 | Hs#S4323355 | g765 | Hs#S4323501 | g975 | Hs#S4324026 |
| g528 | Hs#S4323264 | g620 | Hs#S4323356 | g771 | Hs#S4323507 | g980 | Hs#S4324031 |
| g530 | Hs#S4323266 | g624 | Hs#S4323360 | g772 | Hs#S4323508 | g981 | Hs#S4324032 |
| g532 | Hs#S4323268 | g630 | Hs#S4323366 | g777 | Hs#S4323513 | g983 | Hs#S4324034 |
| g534 | Hs#S4323270 | g641 | Hs#S4323377 | g779 | Hs#S4323515 | g996 | Hs#S4324047 |
| g536 | Hs#S4323272 | g643 | Hs#S4323379 | g780 | Hs#S4323516 | g1023 | Hs#S4324074 |
| g538 | Hs#S4323274 | g645 | Hs#S4323381 | g783 | Hs#S4323519 | g1027 | Hs#S4324078 |
| g543 | Hs#S4323279 | g647 | Hs#S4323383 | g784 | Hs#S4323520 | g1028 | Hs#S4324079 |
| g546 | Hs#S4323282 | g650 | Hs#S4323386 | g788 | Hs#S4323524 | g1031 | Hs#S4324082 |
| g547 | Hs#S4323283 | g652 | Hs#S4323388 | g793 | Hs#S4323529 | g1033 | Hs#S4324084 |
| g548 | Hs#S4323284 | g656 | Hs#S4323392 | g801 | Hs#S4323537 | g1039 | Hs#S4324090 |
| g550 | Hs#S4323286 | g662 | Hs#S4323398 | g804 | Hs#S4323540 | g1046 | Hs#S4324097 |
| g556 | Hs#S4323292 | g665 | Hs#S4323401 | g811 | Hs#S4323547 | g1052 | Hs#S4324103 |
| g564 | Hs#S4323300 | g666 | Hs#S4323402 | g824 | Hs#S4323875 | g1060 | Hs#S4324111 |
| g565 | Hs#S4323301 | g668 | Hs#S4323404 | g826 | Hs#S4323877 | g1063 | Hs#S4324114 |
| g568 | Hs#S4323304 | g671 | Hs#S4323407 | g828 | Hs#S4323879 | g1073 | Hs#S4324124 |
| g569 | Hs#S4323305 | g674 | Hs#S4323410 | g830 | Hs#S4323881 | g1074 | Hs#S4324125 |
| g574 | Hs#S4323310 | g675 | Hs#S4323411 | g831 | Hs#S4323882 | g1075 | Hs#S4324126 |
| g577 | Hs#S4323313 | g683 | Hs#S4323419 | g833 | Hs#S4323884 |  |  |
| g579 | Hs#S4323315 | g684 | Hs#S4323420 | g841 | Hs#S4323892 |  |  |

^a^The item ‘No.’ refers to its index in this work, and ‘Gene ID’ is extracted from the original cDNA library identified by ‘NCI_CGAP_DHO’. See the text.


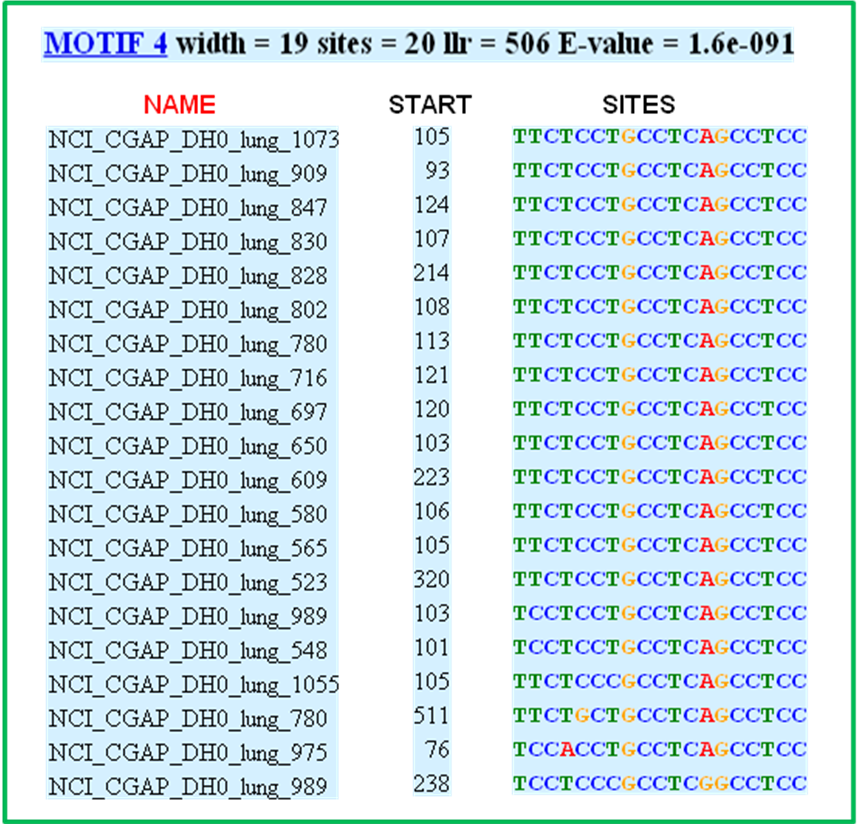

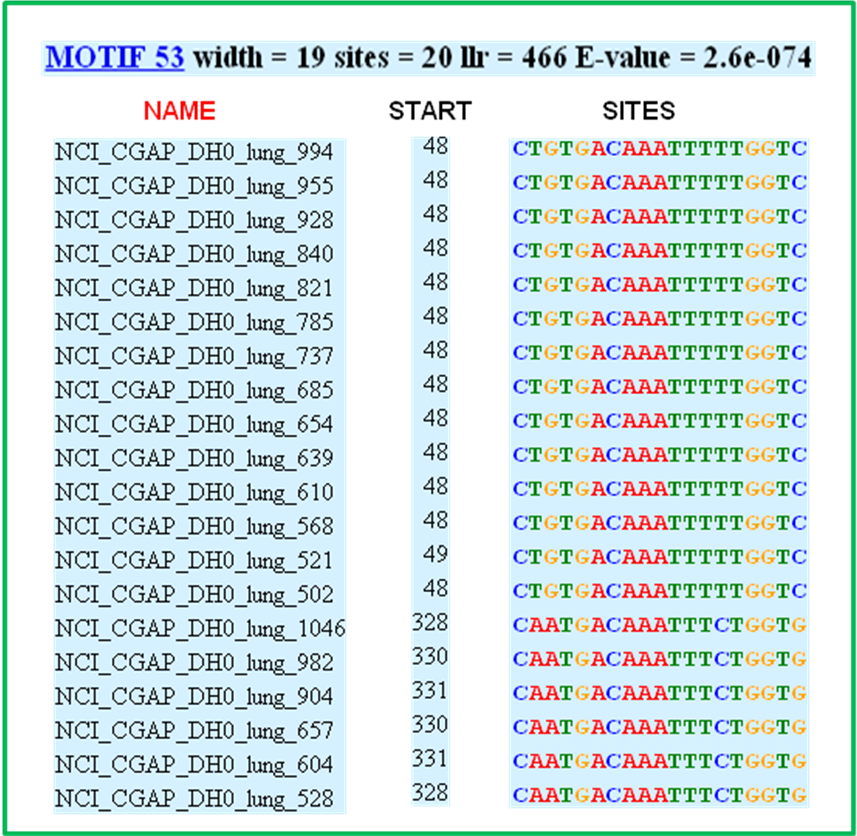


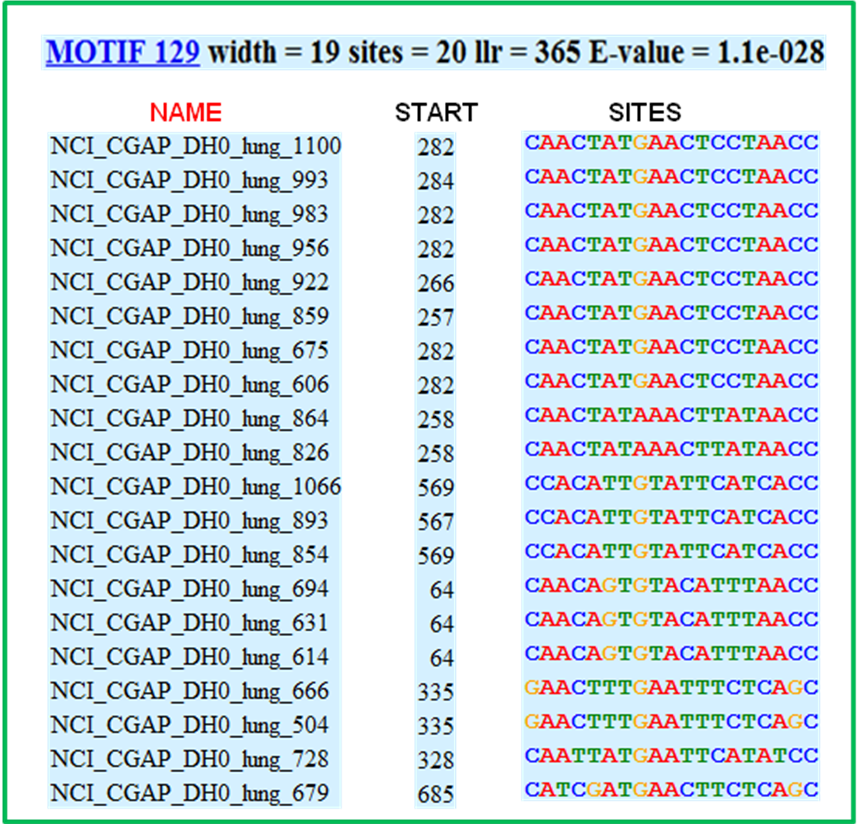

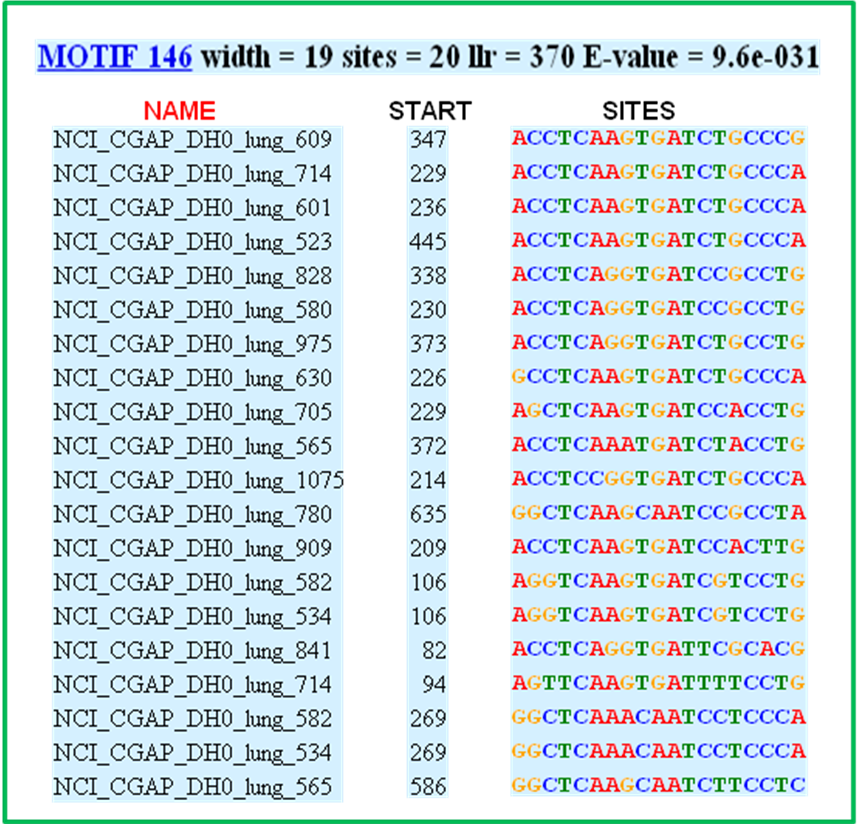


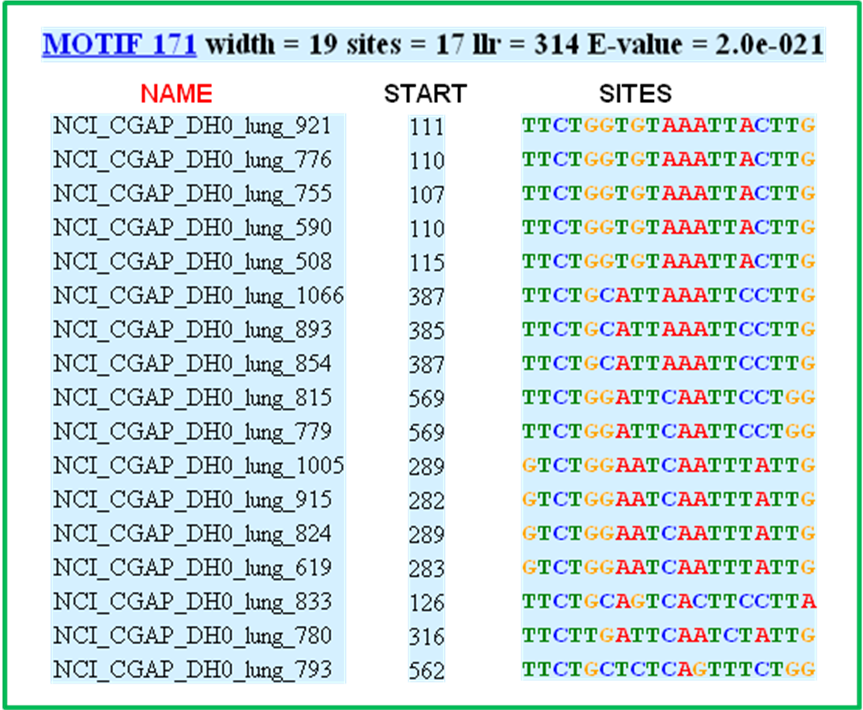

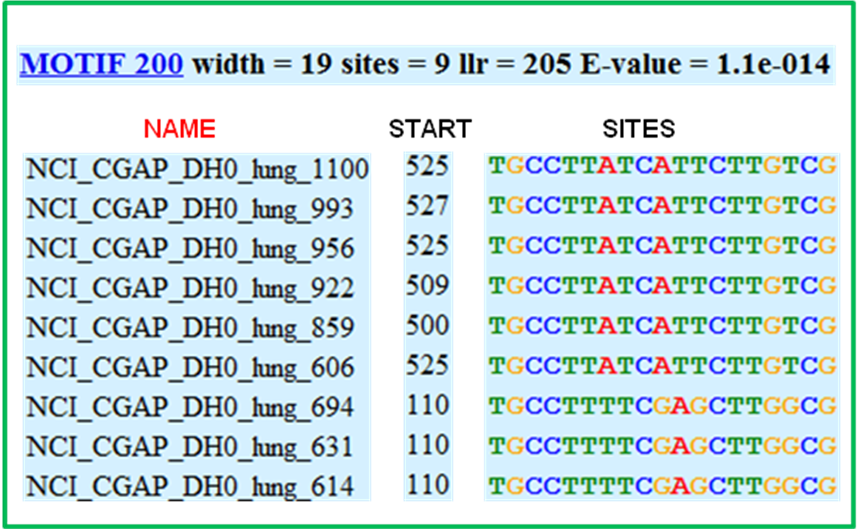


**Figure S7** Examples of the motif search result. The panels refer to six motifs corresponding to s4,s53, s129, s171 and s200 in Table S17 respectively. They are roughly uniformly distributed in the final selected siRNA set (Table S17).


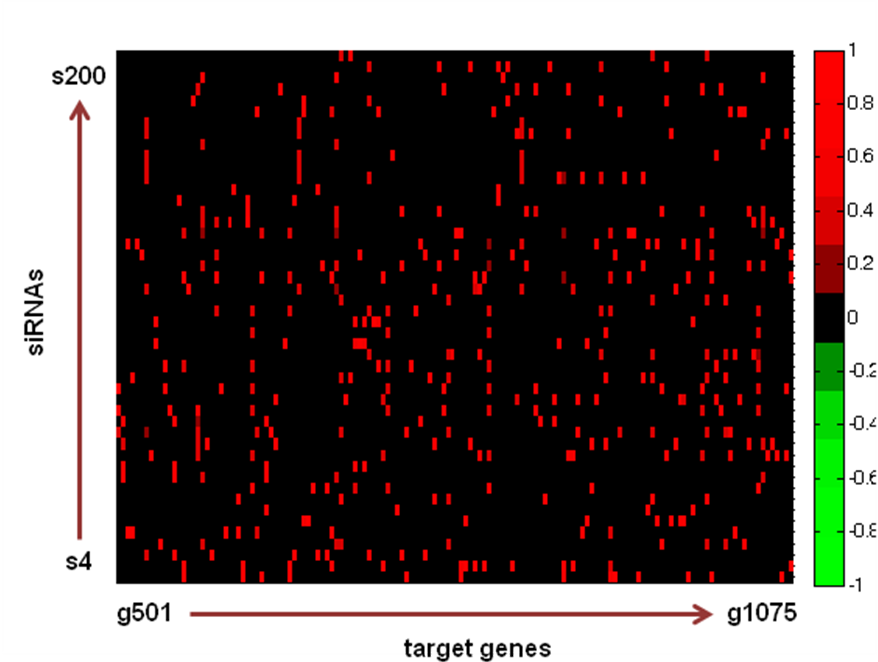


**Figure S8** Heatmap visualization of the CS matrix related to the final selected 48 siRNAs (column) and 146 target genes (row) as listed in Tables S17 and S18 respectively. A red pixel means a significant binding affinity between the corresponding siRNA-target gene pair. Black pixels refer to no affinity. One can tell the sparsity of the CS matrix from the heatmap figure.

**Supplemental References**

1. Huesken D, Lange J, Mickanin C, Weiler J, Asselbergs F, et al. (2005) Design of a genome-wide siRNA library using an artificial neural network. Nat Biotechnol 23: 995-1001.

2. Reynolds A, Leake D, Boese Q, Scaringe S, Marshall WS, et al. (2004) Rational siRNA design for RNA interference. Nat Biotechnol 22: 326-330.

3. Xia T, SantaLucia J, Burkard ME, Kierzek R, Schroeder SJ, et al. (1998) Thermodynamic Parameters for an Expanded Nearest-Neighbor Model for Formation of RNA Duplexes with Watson−Crick Base Pairs†. Biochemistry 37: 14719-14735.

4. Wang J, Zhou XB, Li FH, Bradley PL, Chang SF, et al. (2009) An image score inference system for RNAi genome-wide screening based on fuzzy mixture regression modeling. Journal of Biomedical Informatics 42: 32-40.

5. Manjunath BS, Ma WY (1996) Texture features for browsing and retrieval of image data. IEEE Transactions on PAMI 18: 837-842.

6. Wang J, Zhou X, Bradley PL, Chang SF, Perrimon N, et al. (2008) Cellular phenotype recognition for high-content RNA interference genome-wide screening. J Biomol Screen 13: 29-39.

7. Proakis JG, Manolakis DG (2000) Digital Signal Processing: Principles, Algorithms, and Applications 3rd edition. Upper Saddle River, NJ: Prentice-Hall.

8. Peek AS, Behlke MA (2007) Design of active small interfering RNAs. Curr Opin Mol Ther 9: 110-118.
